# Supplementary figures and images for: MED19 alters AR occupancy and gene expression in prostate cancer cells, driving MAOA expression and growth under low androgen
Source: PLoS Genet. 2021 Jan 29;17(1):e1008540. doi: 10.1371/journal.pgen.1008540 (PMC7875385; doi:10.1371/journal.pgen.1008540)

# S1 Fig

A

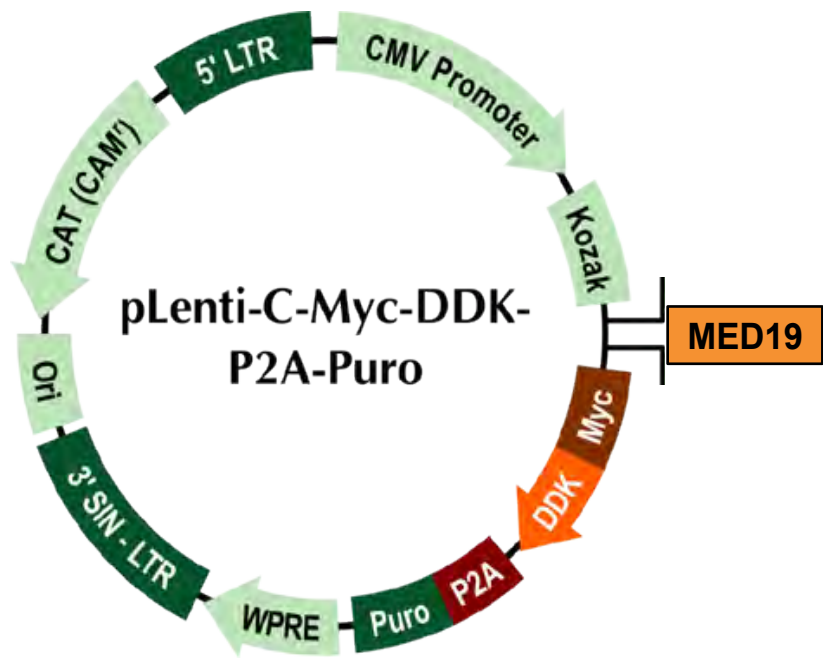

B

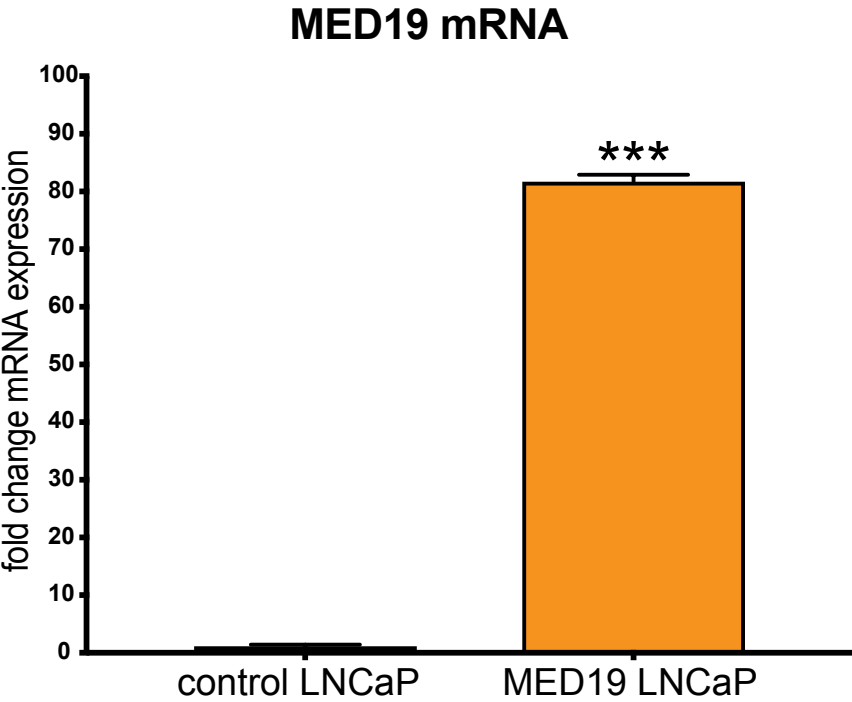

C

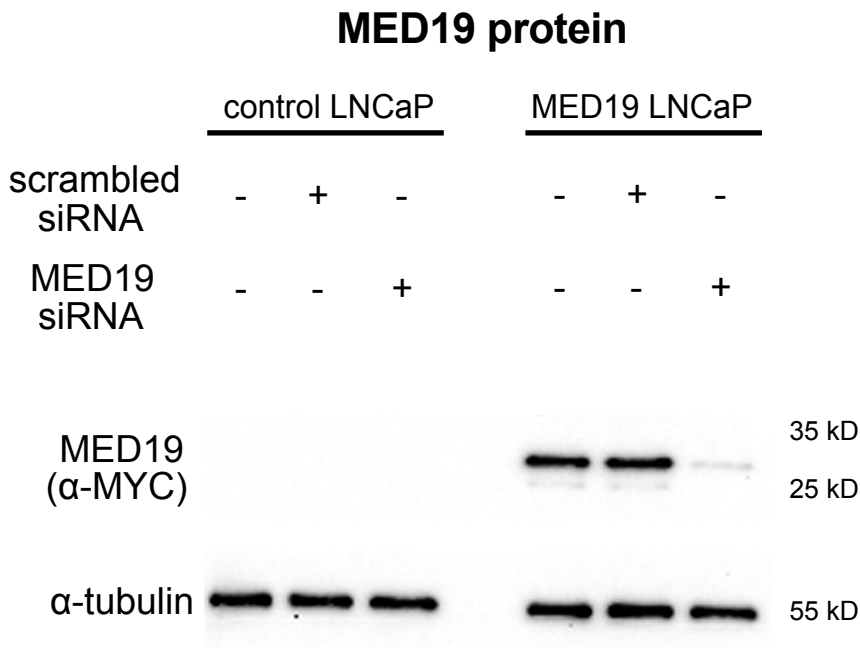

D

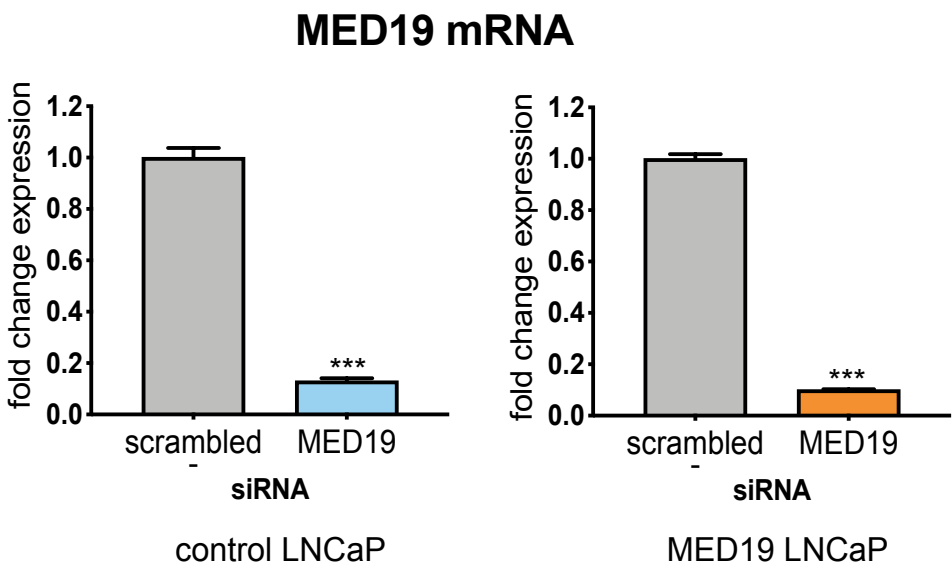

Supplement: S1 Fig — MED19 LNCaP cells with stable overexpression of MYC- and FLAG-tagged MED19 and control LNCaP cells with stable expression of the empty vector were created by lentiviral transduction, with pooled clones selected with puromycin. After selection, stable overexpression of MED19 in MED19 LNCaP cells was confirmed. A) Scheme of the lentiviral expression construct MED19 (NM_153450) (adapted from Origene). B) RNA was extracted and qPCR was performed for MED19 in control LNCaP cells and MED19 LNCaP cells to confirm upregulation of MED19 mRNA (fold change expression normalized to RPL19 with MED19 mRNA expression in control LNCaP cells set as “1”). ***p < 0.001. C) MED19 LNCaP cells and control LNCaP cells were treated with MED19 siRNA or scrambled siRNA, and total protein lysates were probed by MYC tag. Tubulin was used as a loading control. D) Validation of MED19 knockdown, with MED19 mRNA measured as in B (with MED19 mRNA expression with scrambled siRNA treatment set as “1”). (PDF) [file pgen.1008540.s001.pdf]

# S2 Fig

A

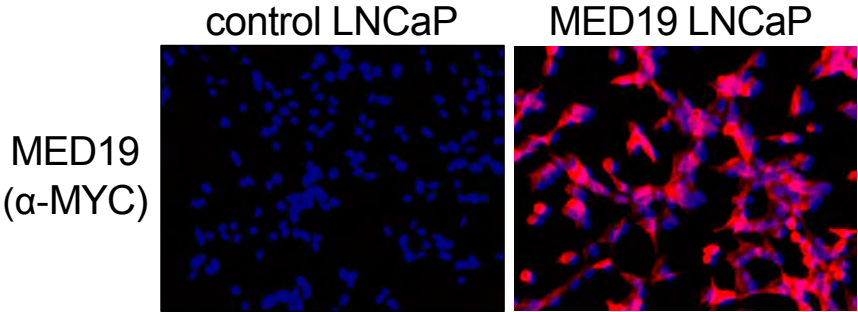

B

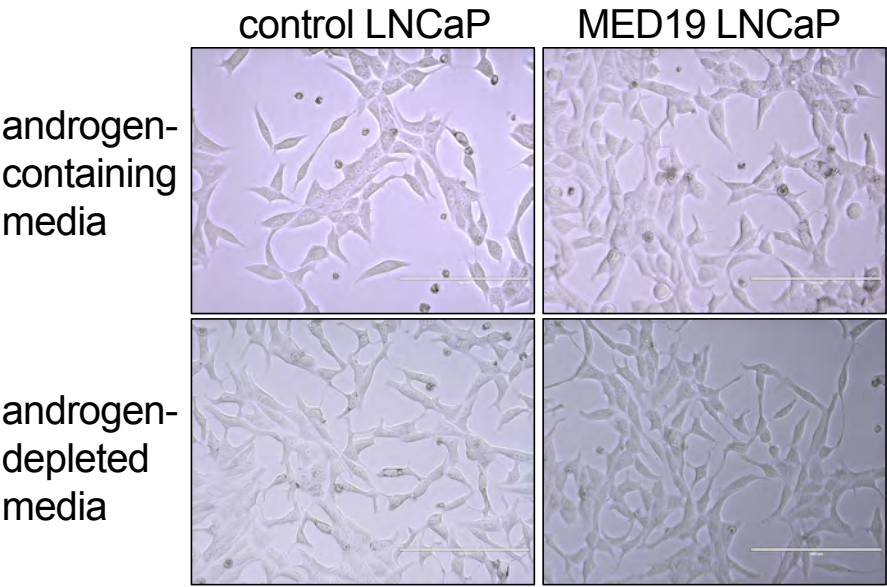

C

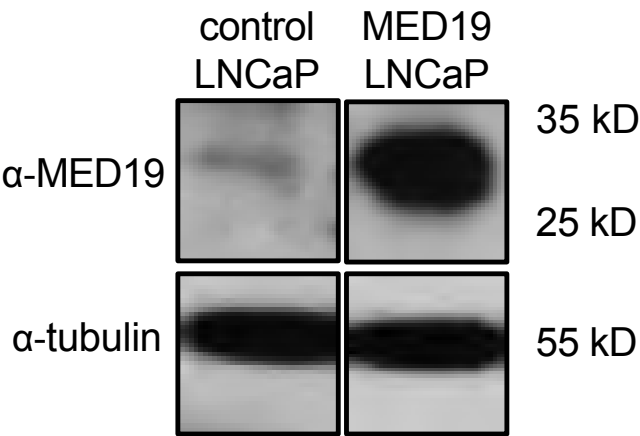

Supplement: S2 Fig — A) Control LNCaP and MED19 LNCaP cells were cultured in complete media, fixed with paraformaldehyde, permeabilized, and stained with a mouse monoclonal antibody to MYC (Myc-Tag (9B11) Cell Signaling #2276), which is an epitope tag on the MED19 expression construct (see S1A Fig), followed by a secondary antibody (Texas Red anti-mouse), along with DAPI to identify the nucleus (blue), with fluorescent images captured using EVOS Cell Imaging System. Shown is 20X magnification. B) Morphology of control LNCaP and MED19 LNCaP cells. Cells were cultured in androgen-containing media and in androgen-depleted media for 3 days, and imaging of live cells was performed using the EVOS Cell Imaging System. Shown are 20X images. C) Western blot of MED19 from control LNCaP and MED19 LNCaP cells using an antibody to MED19 (developed in our laboratory) that recognizes the endogenous and overexpressed MED19. Tubulin serves as a loading control. (PDF) [file pgen.1008540.s002.pdf]

# S3 Fig

## MED19 protein

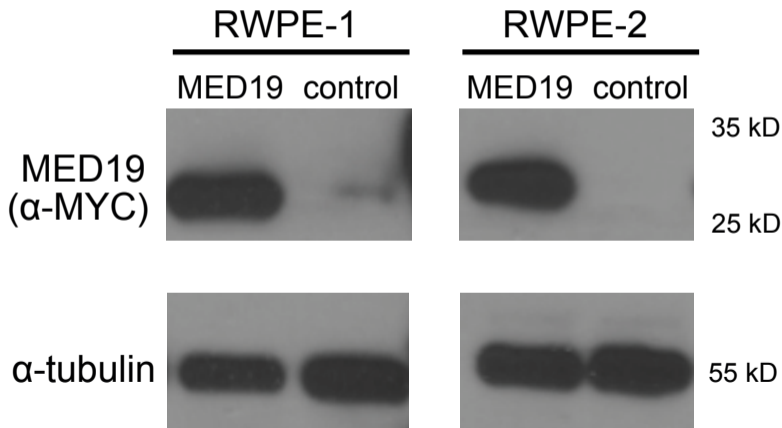

Supplement: S3 Fig — Total protein lysates from RWPE-1 and RWPE-2 cells stably expressing FLAG- and MYC-tagged MED19 (MED19 RWPE-1 and MED19 RWPE-2) or empty vector (control RWPE-1 and control RWPE-2) were probed for MYC tag, with tubulin used as a loading control. (PDF) [file pgen.1008540.s003.pdf]

**S5 Fig**

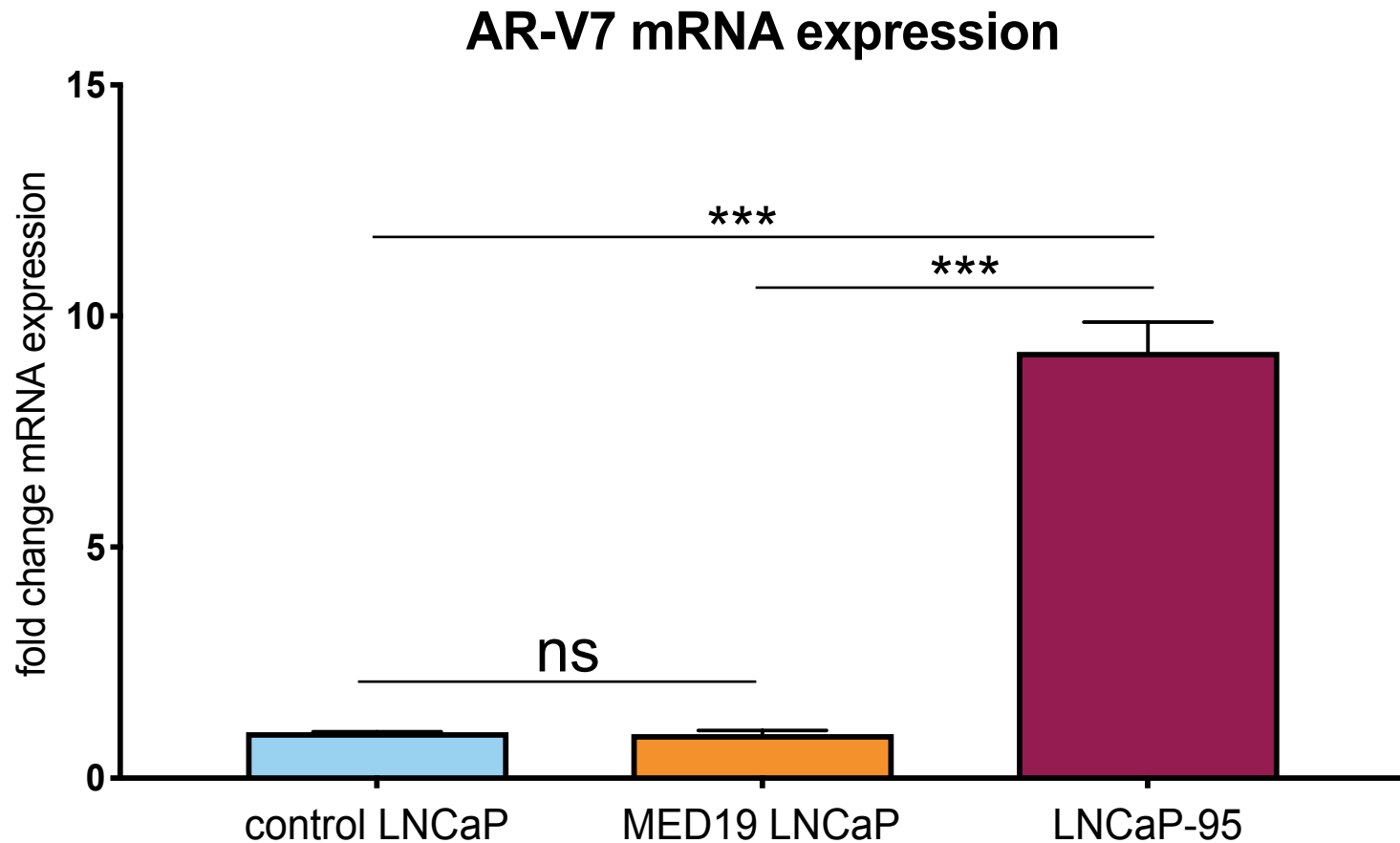

Supplement: S5 Fig — MED19 LNCaP cells and control LNCaP cells were cultured under androgen deprivation for 3 days and treated overnight with ethanol vehicle. RNA was extracted and mRNA measured by qPCR for AR-V7 mRNA (fold change expression normalized to RPL19 with AR-V7 mRNA expression in control LNCaP cells set as “1”). LNCaP-95 cells that express AR-V7 were used as a positive control. *p < 0.05; **p < 0.01; and ***p < 0.001. ns = not significant. (PDF) [file pgen.1008540.s005.pdf]

# S6 Fig

A

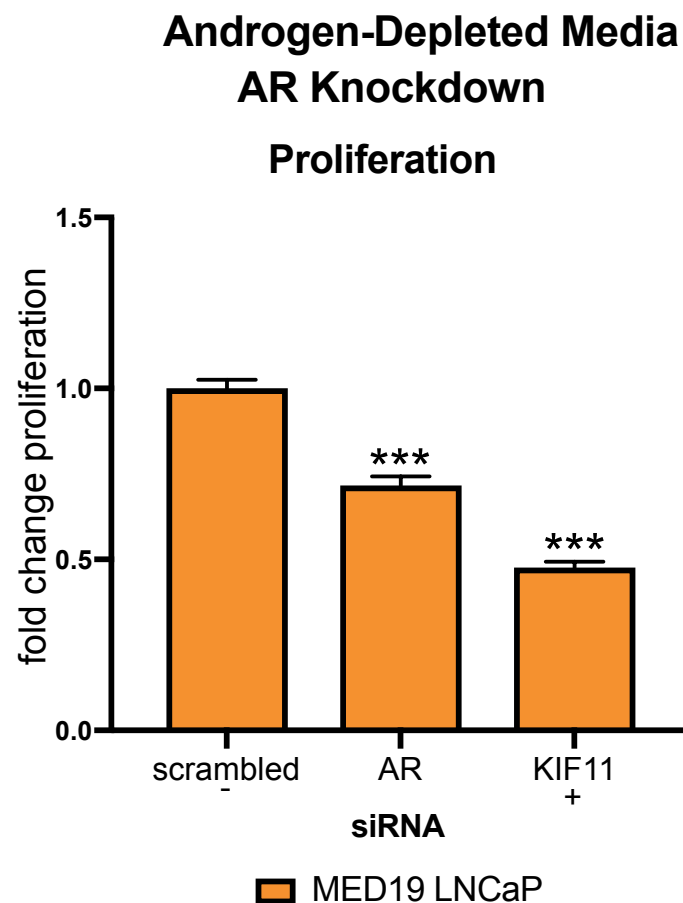

B

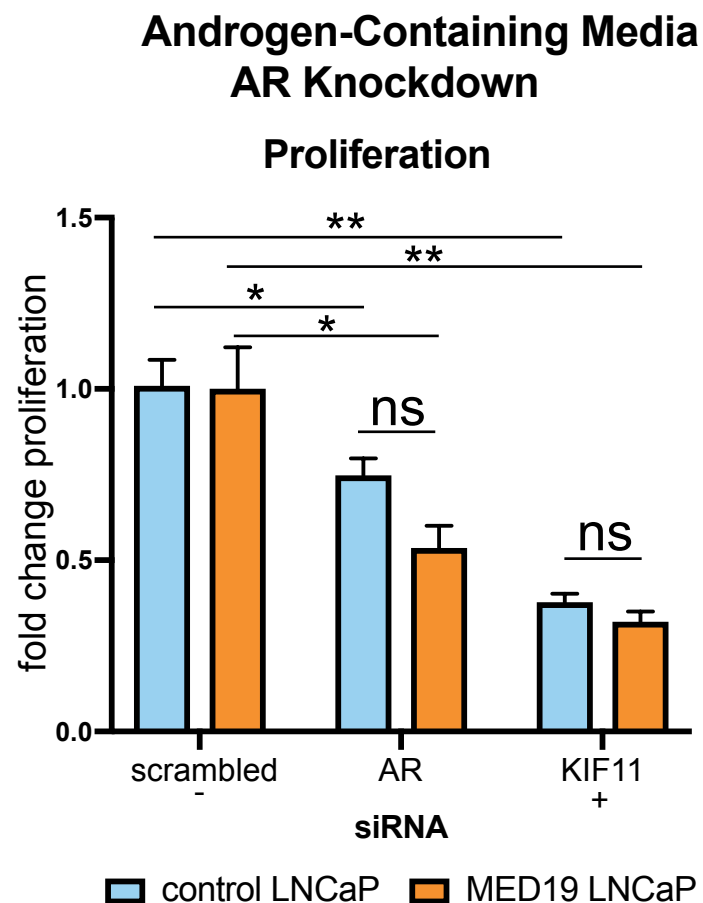

C

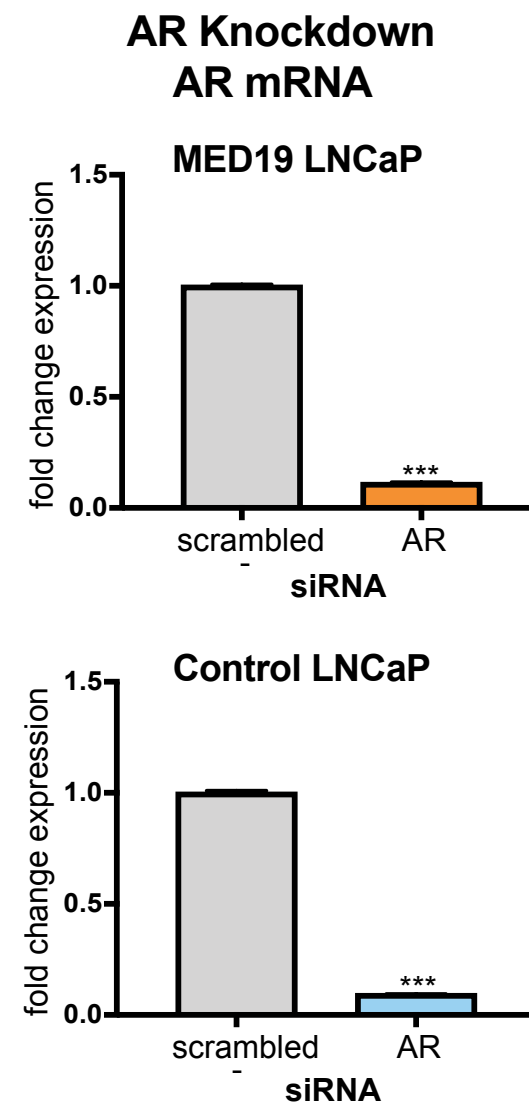

Supplement: S6 Fig — MED19 LNCaP cells were cultured in A) androgen-depleted media or B) androgen-containing media, with control LNCaP cells. AR was depleted by siRNA and proliferation was evaluated after 7 days, normalized to proliferation with scrambled siRNA. KIF11 was used as a positive control. Experiment was performed in biological duplicate, with representative results shown. *p < 0.05; **p < 0.01; and ***p < 0.001. ns = not significant. C) Validation of AR knockdown (fold change expression normalized to RPL19 and AR mRNA expression with scrambled siRNA treatment set as “1”). (PDF) [file pgen.1008540.s006.pdf]

S8 Fig

A

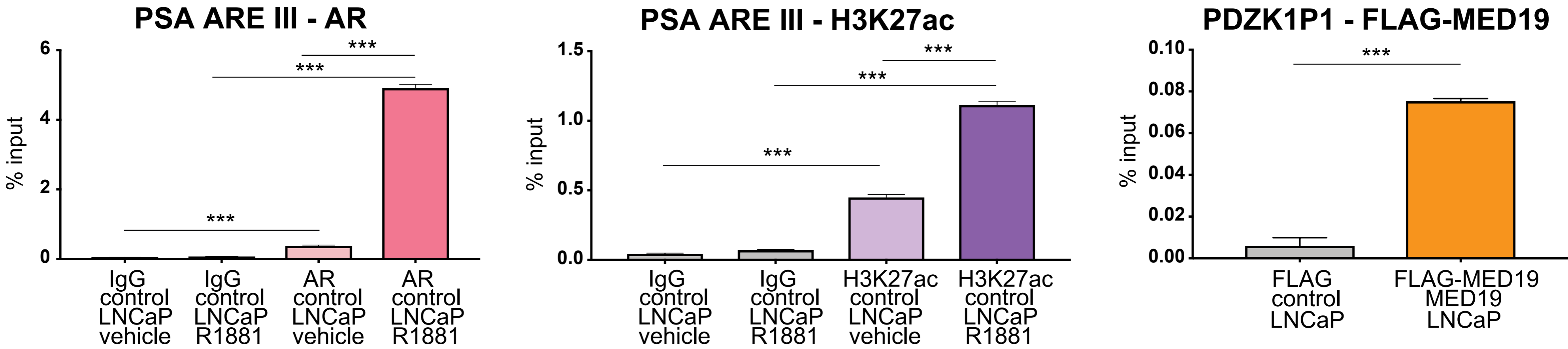

B

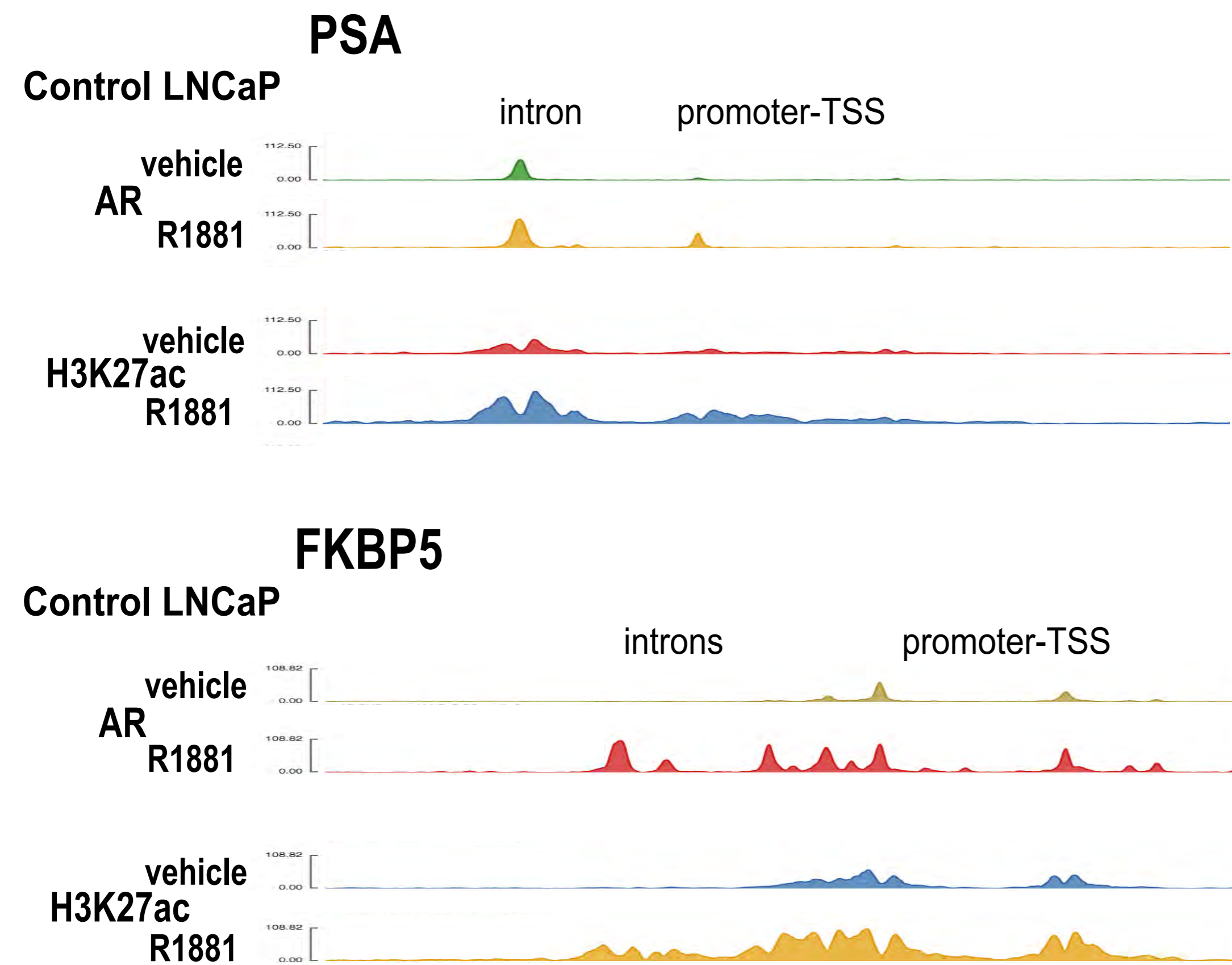

C

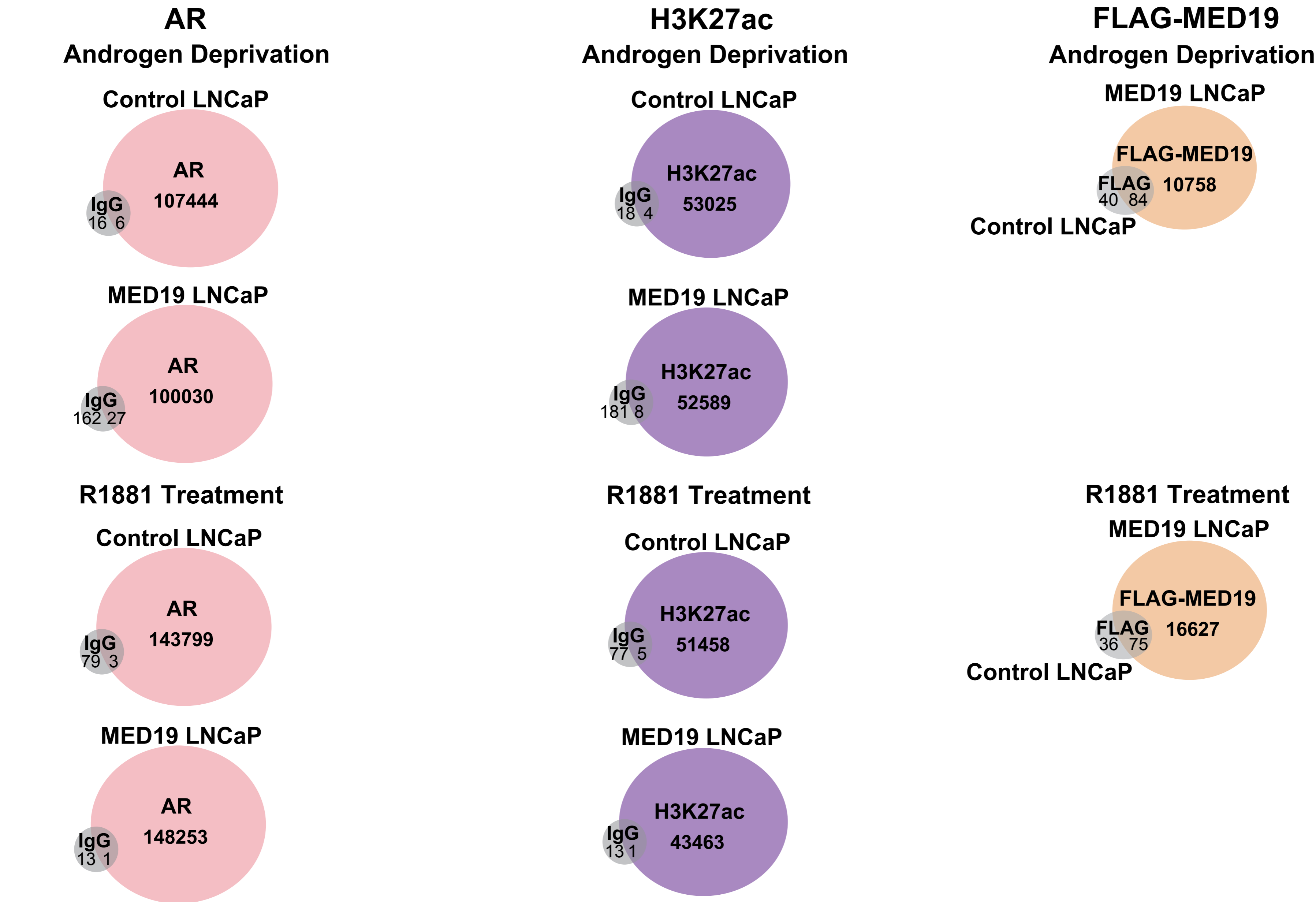

Supplement: S8 Fig — MED19 LNCaP cells and control LNCaP cells were cultured under androgen deprivation for 3 days and treated with ethanol vehicle or 100 nM R1881 for 4 h. ChIP-seq for FLAG-MED19, AR, and H3K27ac was performed in biological triplicate with the exception of ChIP-seq for AR in control LNCaP cells + R1881, where one sample was excluded from the analyses because of low signal. A) ChIP-qPCR QC of AR, H3K27ac, and FLAG-MED19 ChIPs are shown, with normalization to inputs. AR occupancy and H3K27ac at PSA ARE III greatly increase in response to R1881 treatment. IgG is shown as a negative control. FLAG-MED19 shows high occupancy in MED19 LNCaP cells at PDZK1P1, identified as a site of strong FLAG-MED19 occupancy from a pilot ChIP-seq for FLAG-MED19. FLAG in control LNCaP cells is shown as a negative control. Experiments were performed in biological triplicate, with representative results shown. *p < 0.05; **p < 0.01; and ***p < 0.001. B) ChIP-seq tracks (representative results) for AR and H3K27ac at PSA and FKBP5 in control LNCaP cells with vehicle or R1881 treatment. AR occupancy and H3K27ac clearly increase in response to R1881 treatment (occupancy scores in S7 Table). C) Overlap of IgG with AR (left) and H3K27ac sites (middle); and overlap of FLAG in control LNCaP cells with FLAG-MED19 in MED19 LNCaP cells (right). All normalized to input. IgG and FLAG-control yield very few sites, with minimal overlap (all sites in S7 Table). (PDF) [file pgen.1008540.s008.pdf]

S9 Fig

A

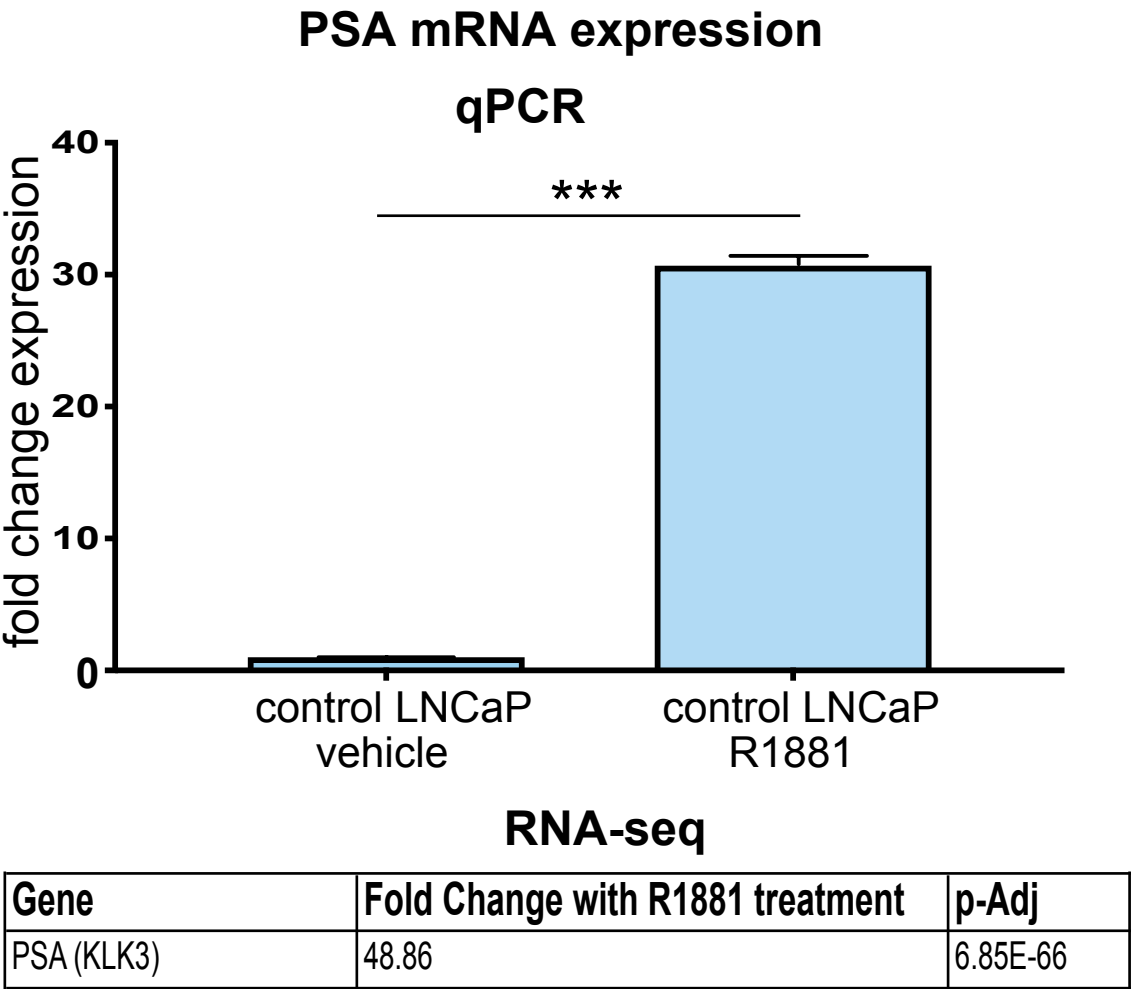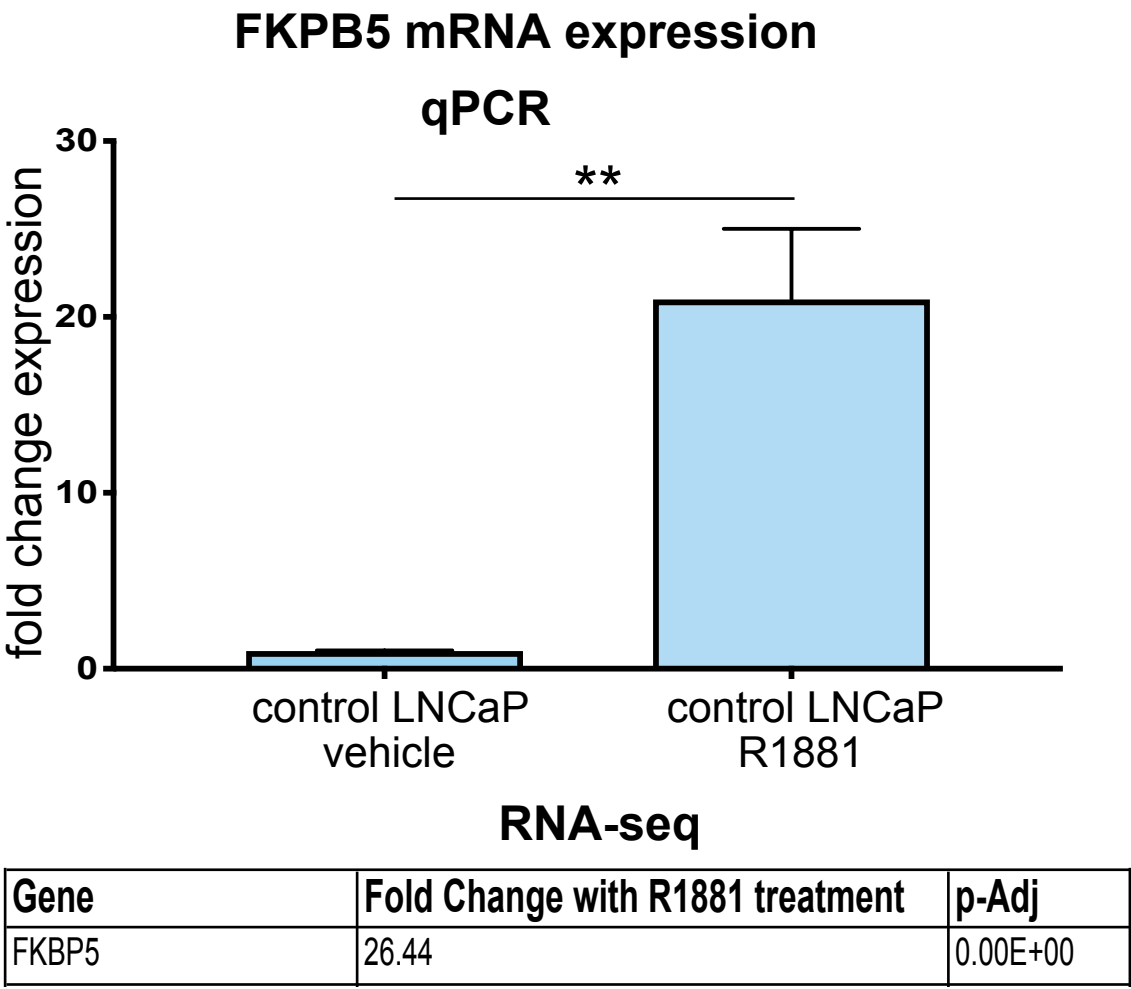

B

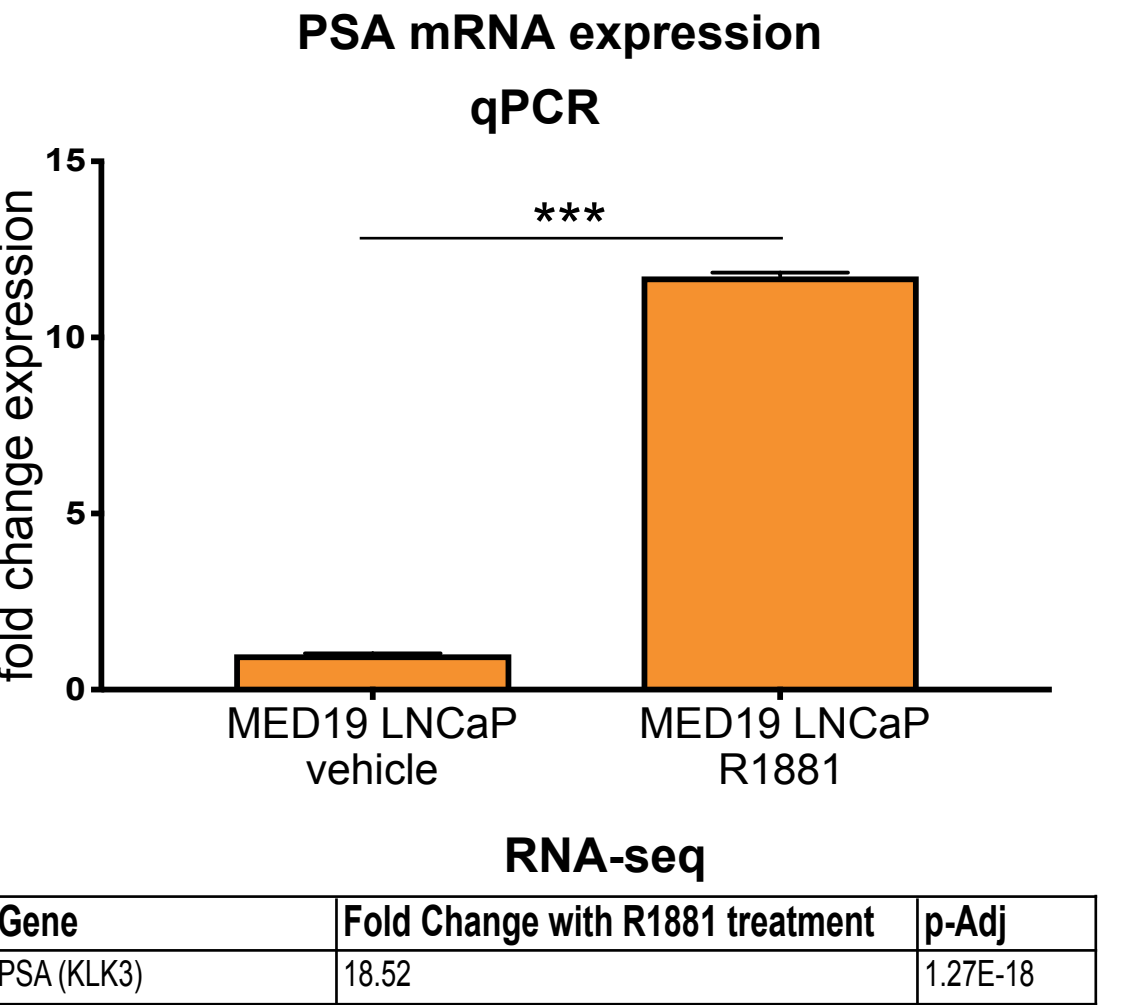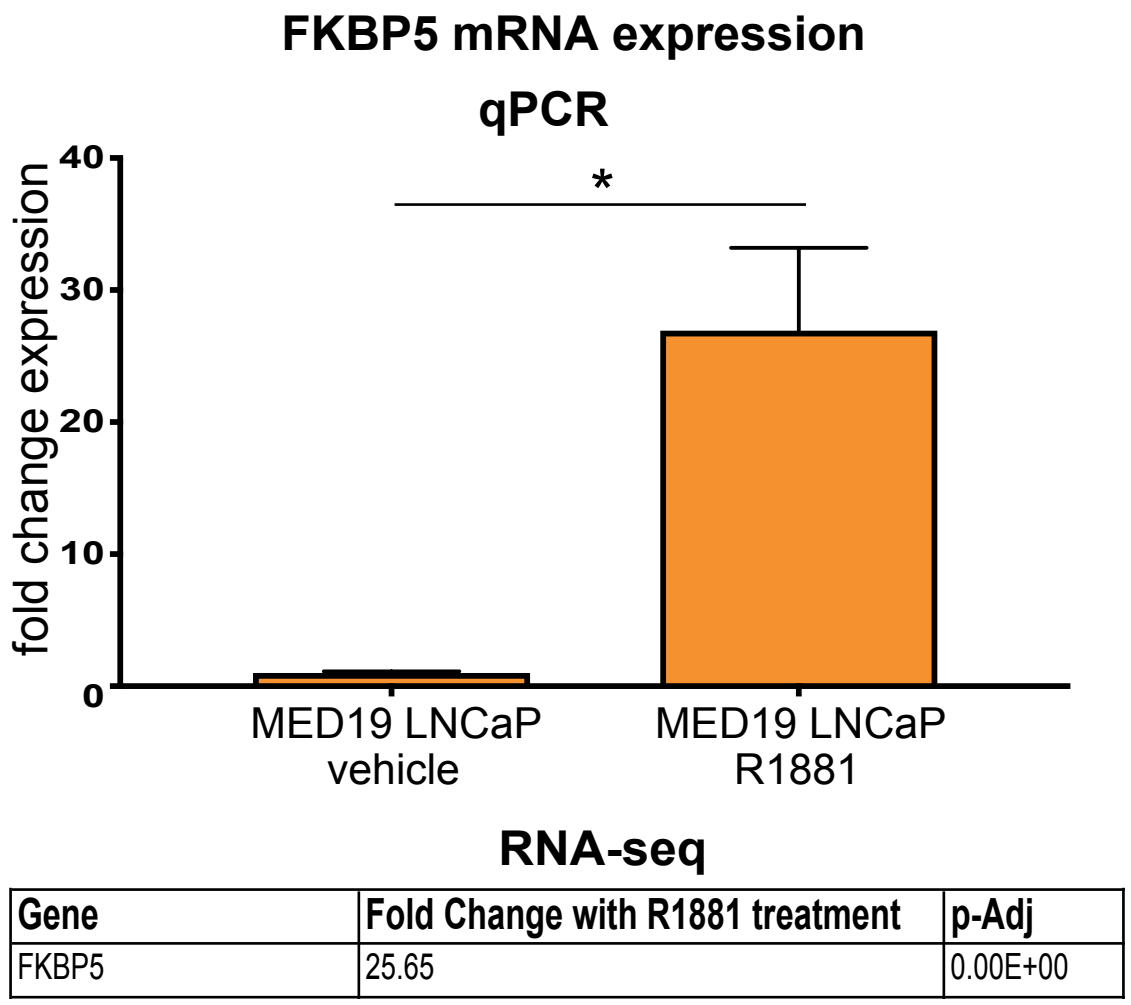

Supplement: S9 Fig — MED19 LNCaP cells and control LNCaP cells were cultured under androgen deprivation for 3 days and treated for 16 h with ethanol vehicle or 10 nM R1881. RNA-seq was performed in biological triplicate. Graphs represent fold changes from qPCR (fold change expression normalized to RPL19 with PSA or FKBP5 mRNA expression in vehicle-treated cells set as “1”) and tables represent fold changes from RNA-seq. Upregulation of PSA and FKBP5 mRNA expression in A) control LNCaP cells and B) MED19 LNCaP cells in response to R1881 treatment, with consistency between RNA-seq and qPCR, and expected increase in expression with R1881 treatment. Experiments were performed in biological triplicate, with representative results shown. *p < 0.05; **p < 0.01; and ***p < 0.001. (PDF) [file pgen.1008540.s009.pdf]

S10 Fig

A

AR sites

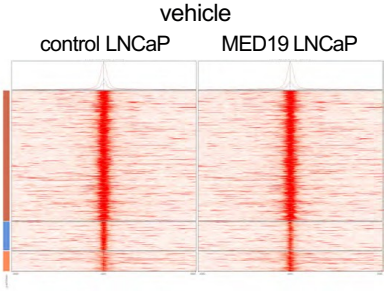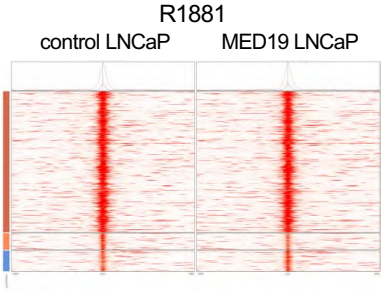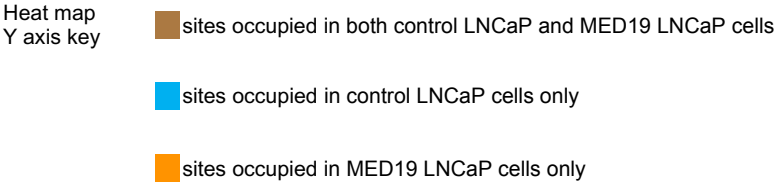

B

H3K27ac sites

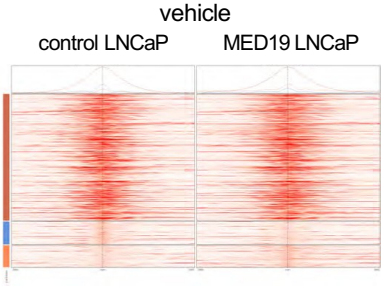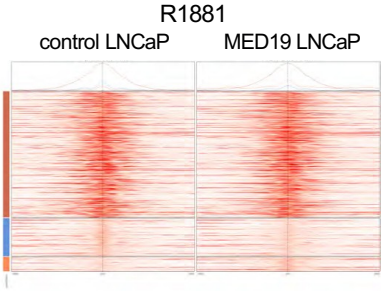

C

AR-FLAG-MED19 sites

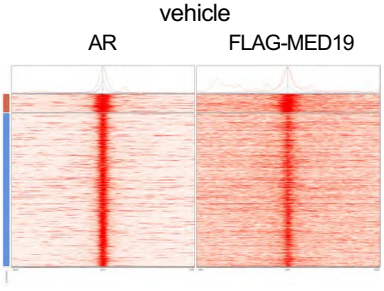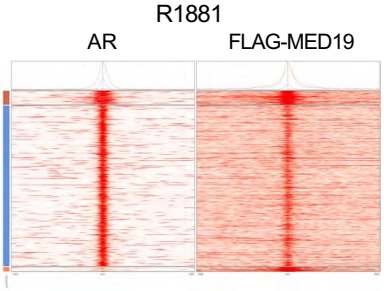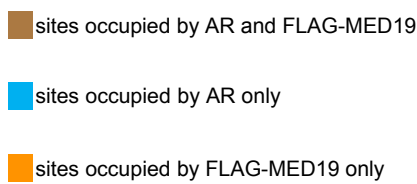

D

AR-FLAG-MED19-H3K27ac sites

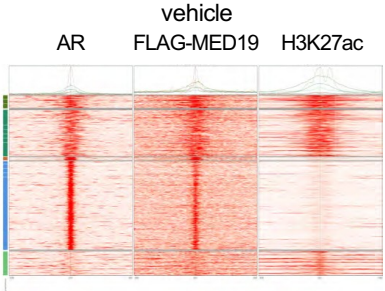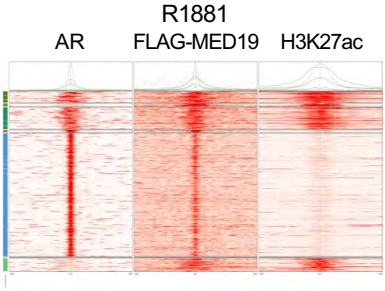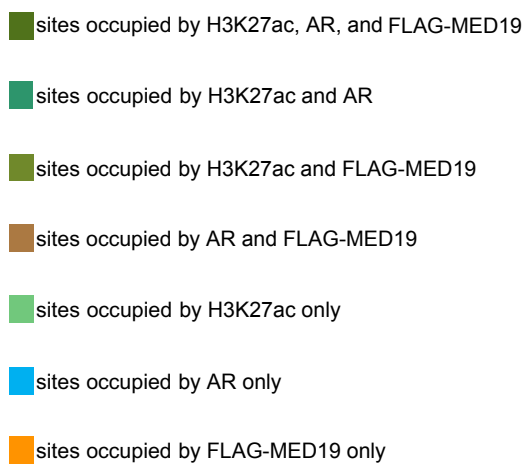

Supplement: S10 Fig — Control LNCaP and MED19 LNCaP cells were cultured under androgen deprivation for 3 days and treated with ethanol vehicle or 100 nM R1881 for 4 hours. ChIP-seq for FLAG-MED19, AR, and H3K27ac were performed and in biological triplicate, with the exception of ChIP-seq for AR in control LNCaP cells + R1881, where one sample was excluded from the analyses because of low signal. A) Binding heatmaps for AR occupancy in control LNCaP and MED19 LNCaP cells. Brown = sites occupied by AR in both control LNCaP and MED19 LNCaP cells; blue = sites occupied by AR in control LNCaP cells only; orange = sites occupied by AR in MED19 LNCaP cells only. B) Binding heatmaps for H3K27 acetylation. Brown = sites of H3K27 acetylation in both control LNCaP and MED19 LNCaP cells; blue = sites of H3K27 acetylation in control LNCaP cells only; orange = sites of H3K27 acetylation in MED19 LNCaP cells only. C) Binding heatmaps for AR and FLAG-MED19 in MED19 LNCaP cells. Brown = sites occupied by AR and FLAG-MED19; blue = sites occupied by AR only; orange = sites occupied by FLAG-MED19 only. D) Binding heatmaps for AR, FLAG-MED19, and H3K27ac in MED19 LNCaP cells. Dark brown-green = sites occupied by AR and FLAG-MED19 and marked by H3K27 acetylation; medium green = sites occupied by AR and marked by H3K27 acetylation; medium brown-green = sites of FLAG-MED19 occupancy and marked by H3K27 acetylation; brown = sites of AR and FLAG-MED19 occupancy; light green = sites of H3K27 acetylation only; blue = sites occupied by AR only; orange = sites occupied by FLAG-MED19 only. (PDF) [file pgen.1008540.s010.pdf]

S17 Fig

A

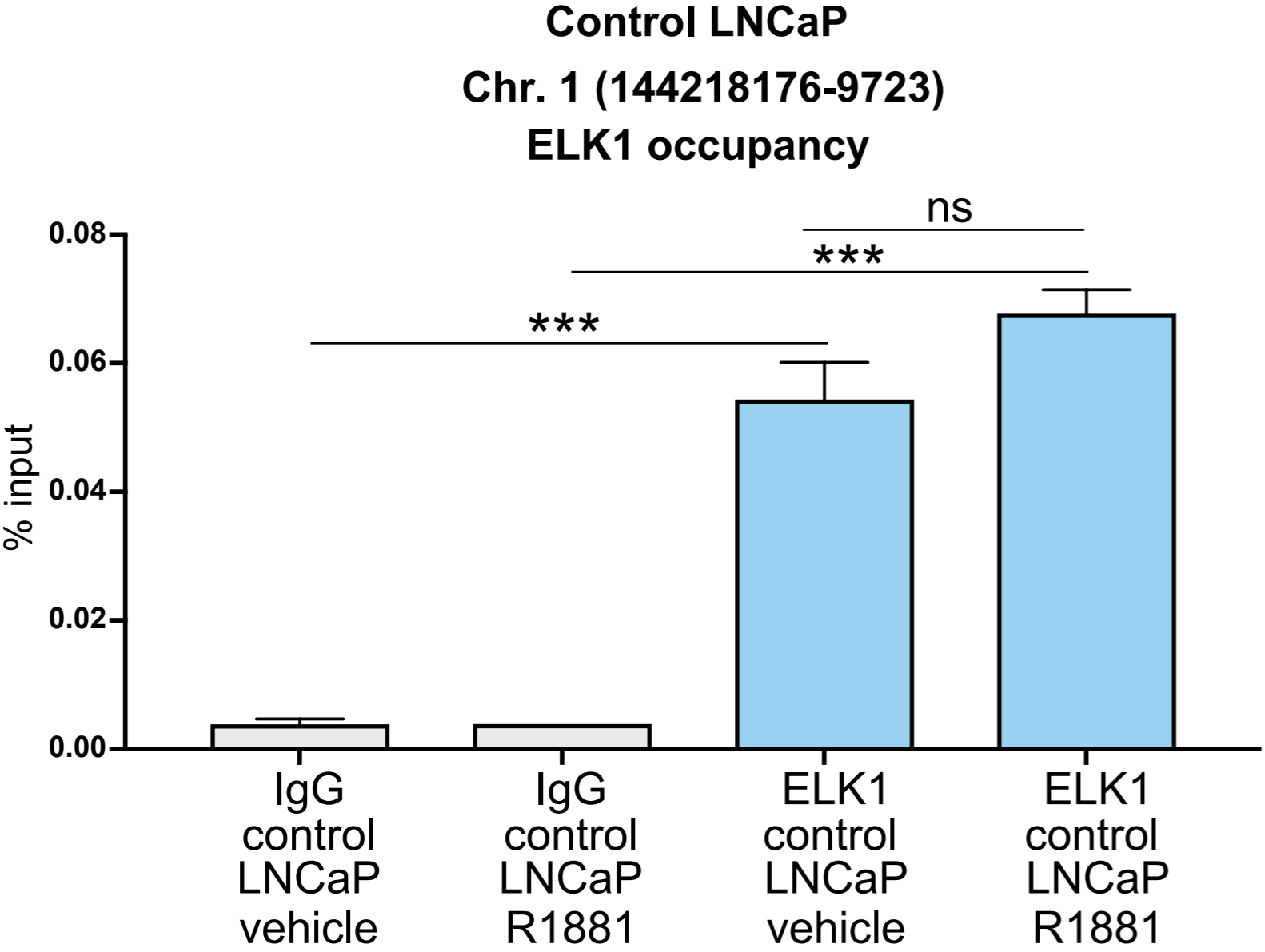

B

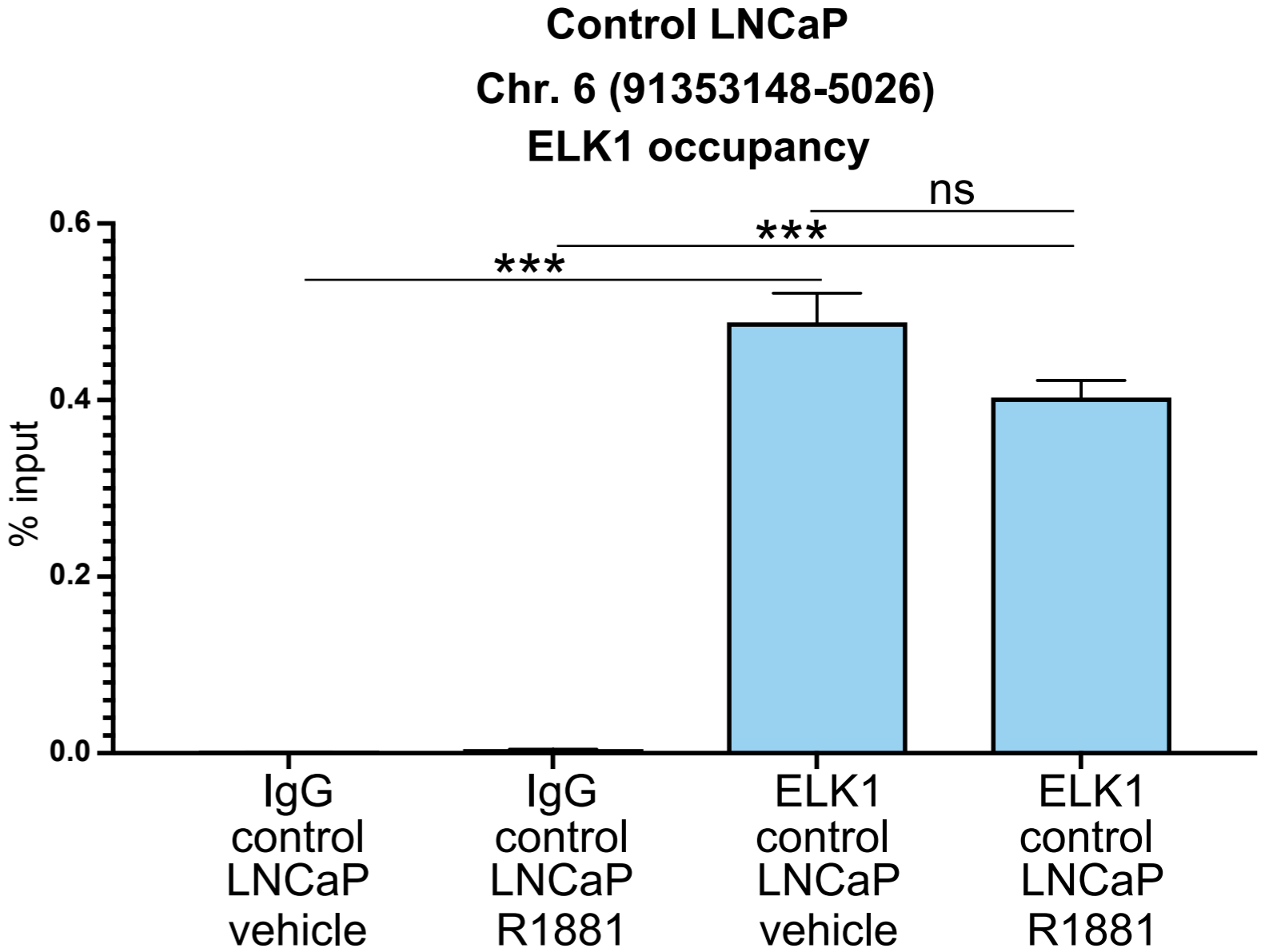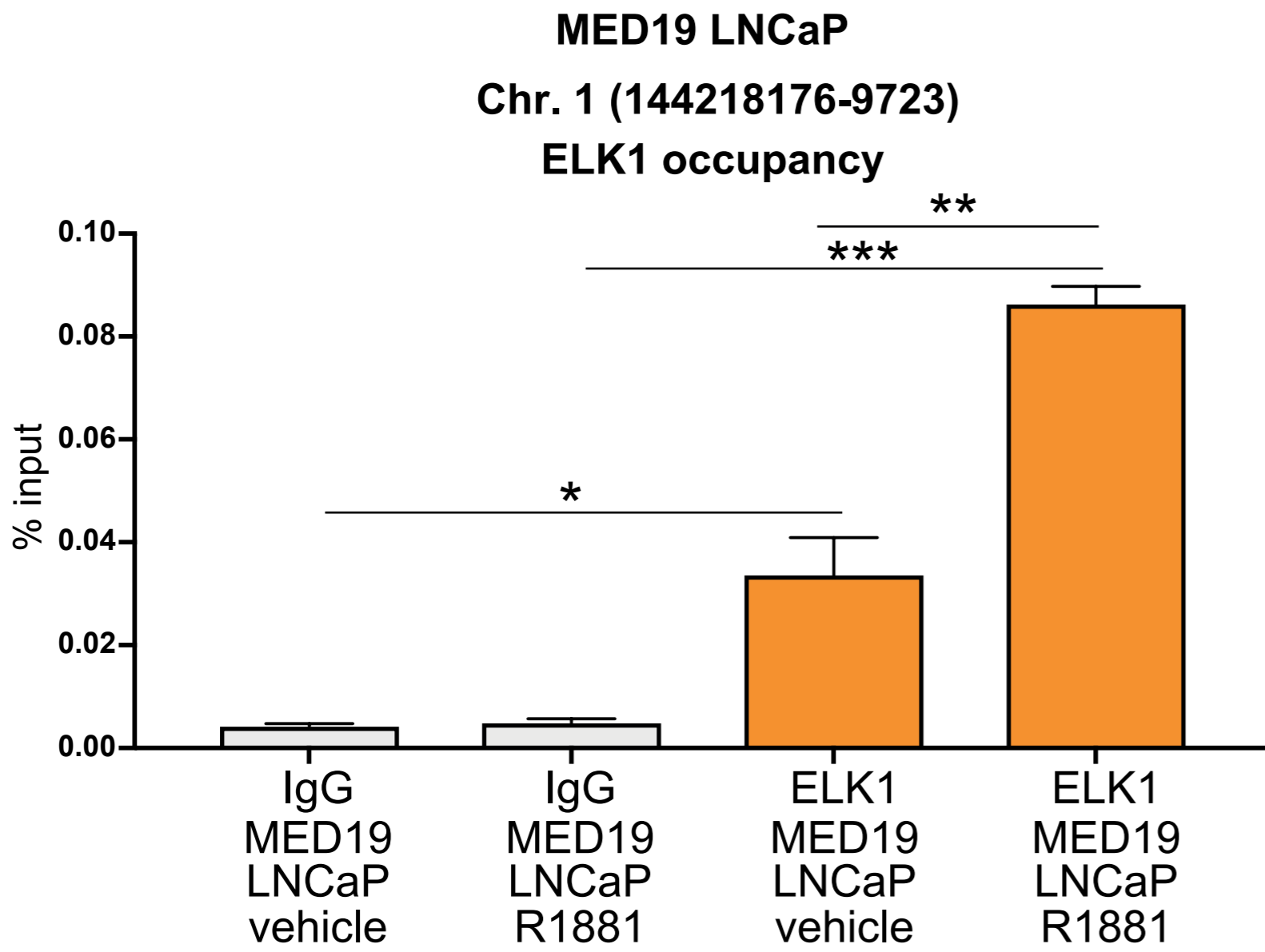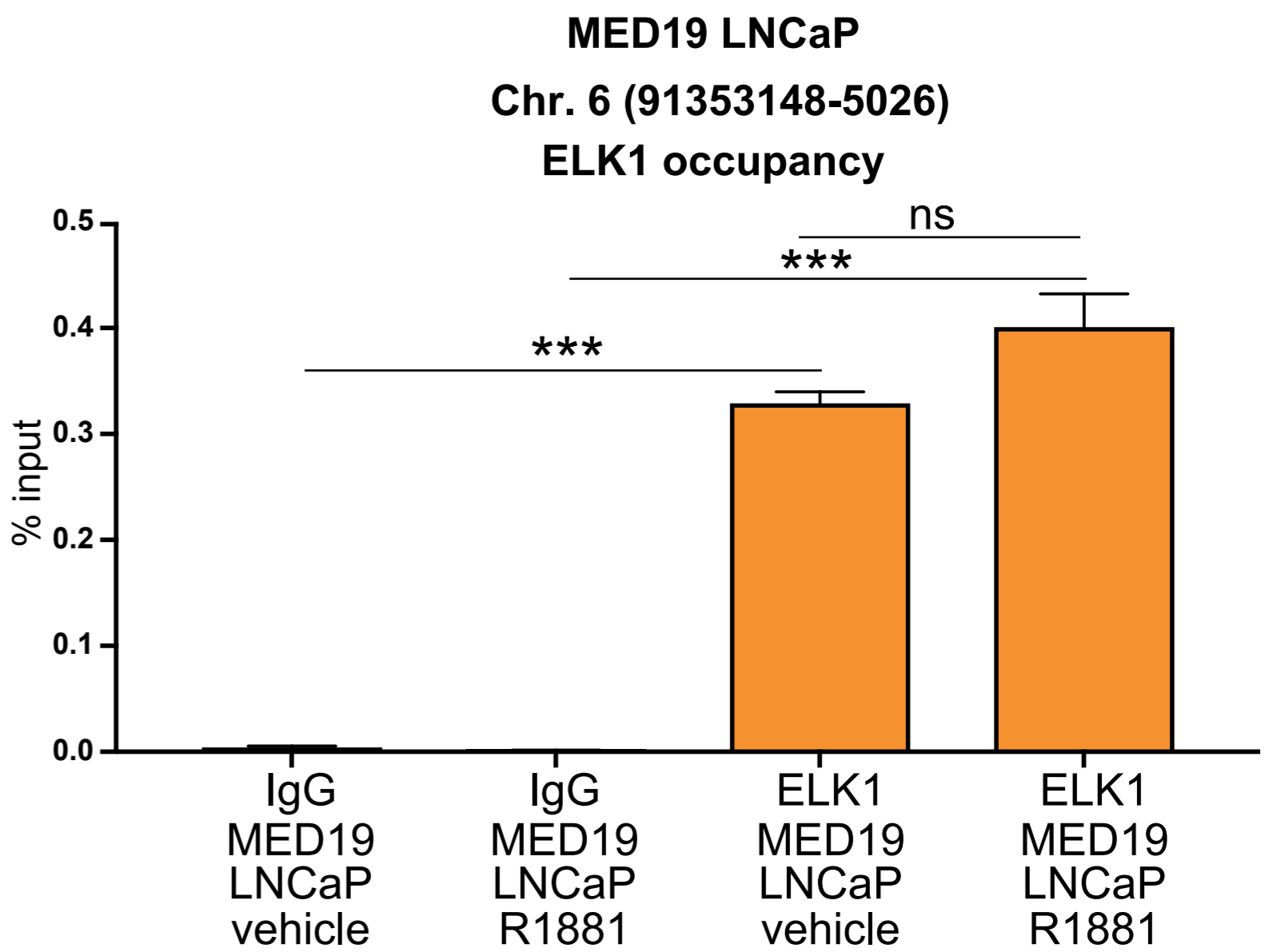

Supplement: S17 Fig — MED19 LNCaP cells and control LNCaP cells were cultured under androgen deprivation for 3 days and treated with ethanol vehicle or 100 nM R1881 for 4 hours, and ChIP-qPCR for ELK1 was performed. ELK1 occupancy at previously published ELK1 sites was verified in control LNCaP cells (top) and MED19 LNCaP cells (bottom), with occupancy verified +/- R1881 for sites at A) Chr.1 and B) Chr. 6. Normalization to inputs was done and IgG is shown as a negative control. *p < 0.05; **p < 0.01; and ***p < 0.001. ns = not significant. (PDF) [file pgen.1008540.s017.pdf]

# S18 Fig

## FLAG-MED19 IP

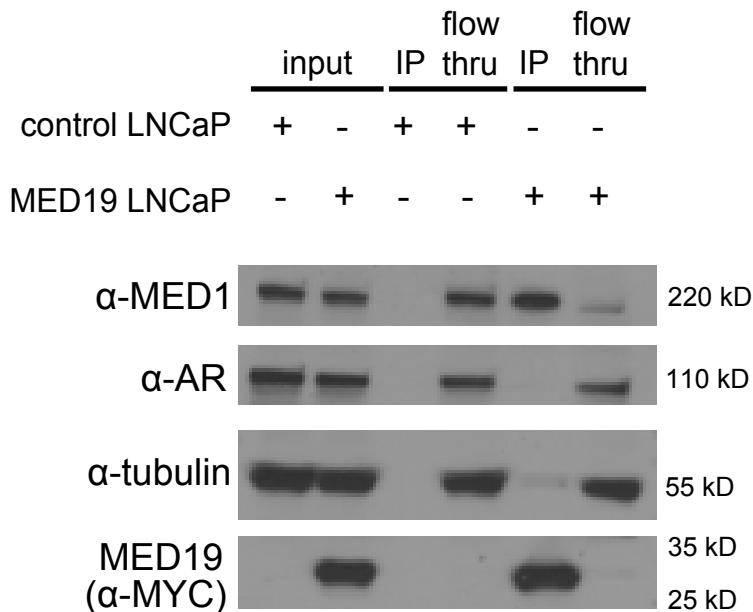

Supplement: S18 Fig — MED19 LNCaP and control LNCaP cells were cultured in androgen-containing media. Cells were lysed with RIPA buffer, input was collected, and FLAG-tagged MED19 was immunoprecipitated using FLAG antibody coupled to protein A/G agarose beads. Total protein lysates (input), FLAG immunoprecipitates, and flow through were collected and probed with antibodies for MYC (for overexpressed MED19), AR, and MED1. Tubulin was used as a loading control for input. Experiment was performed in biological duplicate, with representative results shown. (PDF) [file pgen.1008540.s018.pdf]

S19 Fig

A

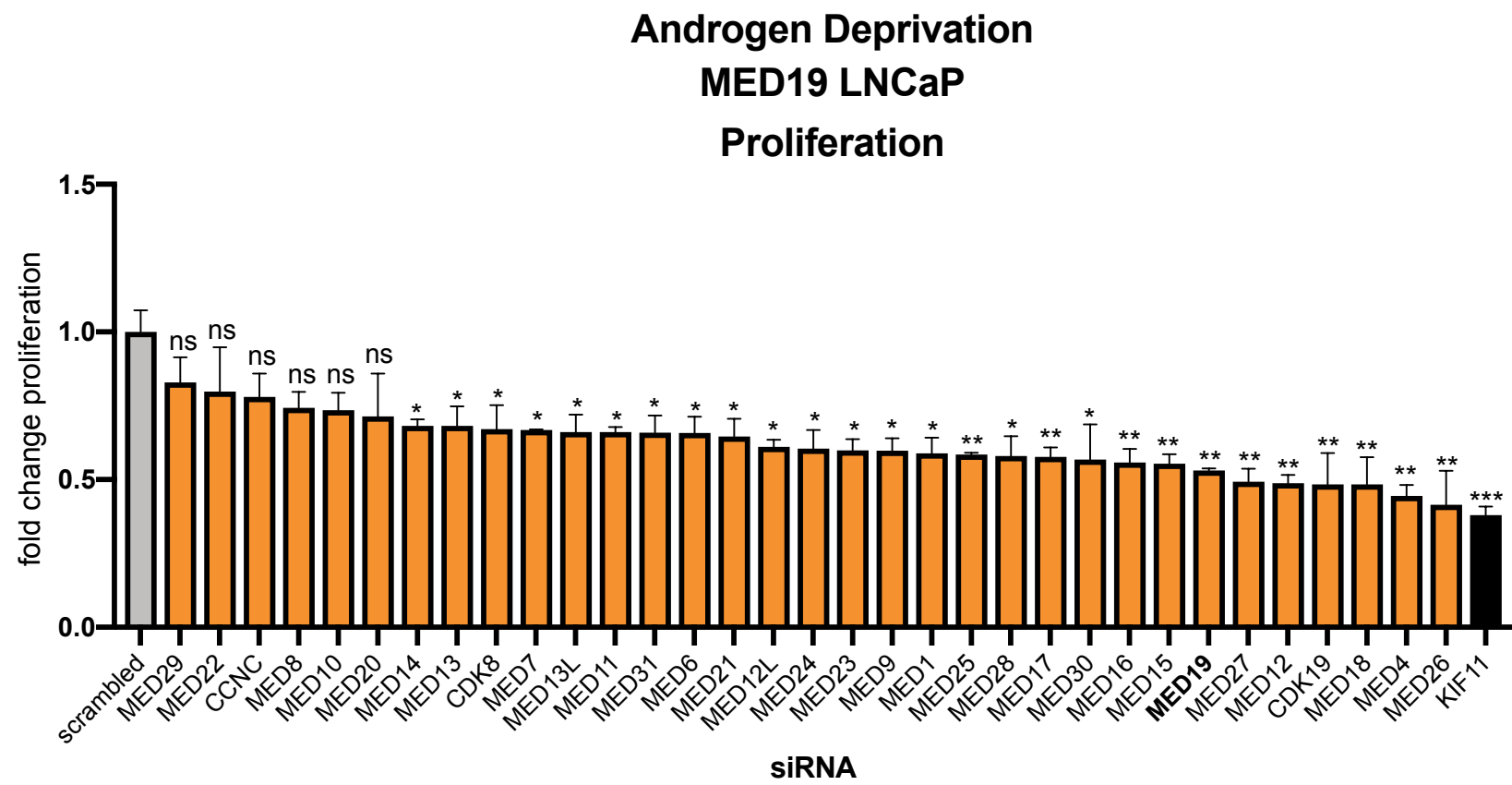

B

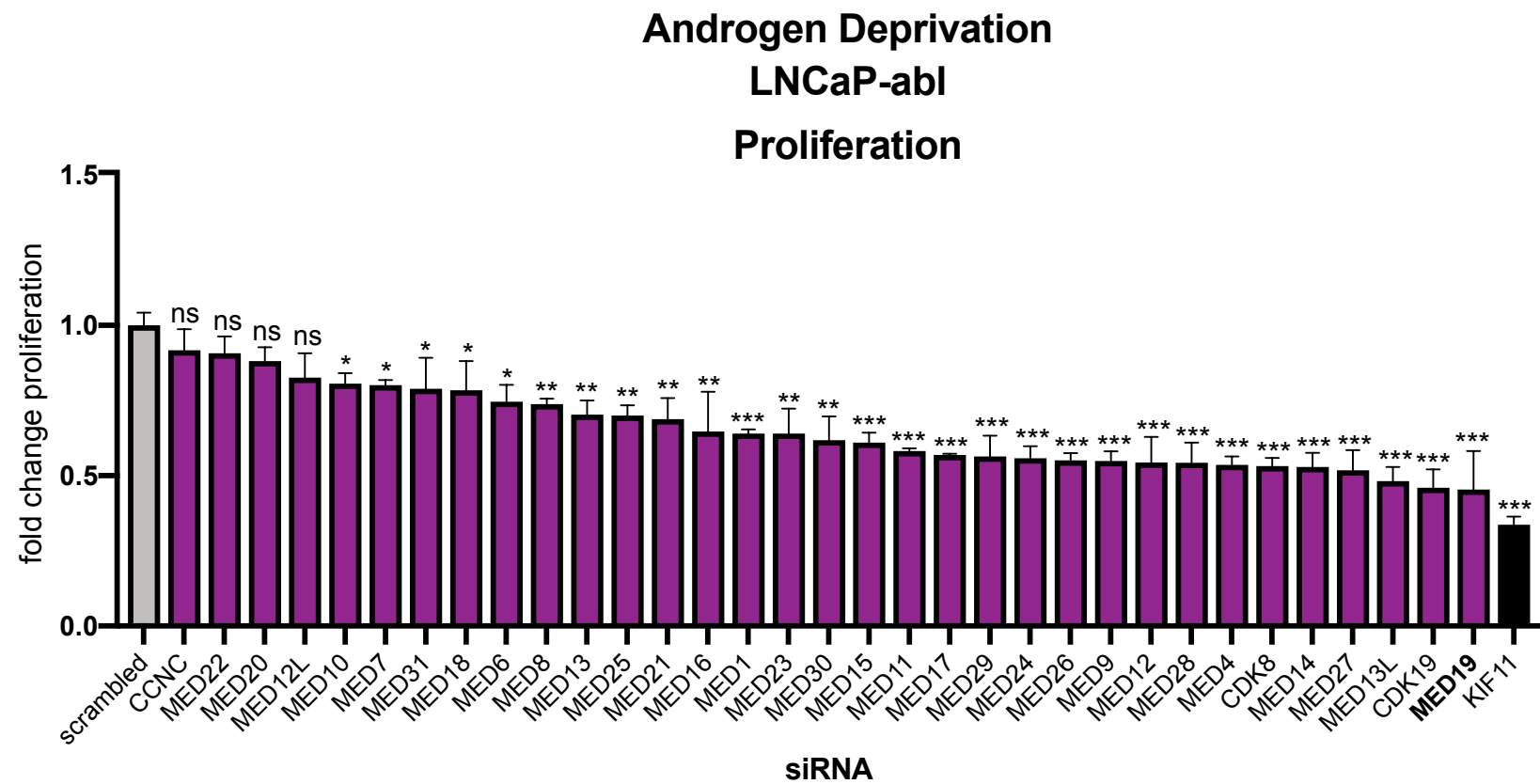

Supplement: S19 Fig — Each Mediator subunit or associated factor from the kinase module was depleted by siRNA and proliferation in androgen-depleted media was evaluated after 5 days, normalized to proliferation with scrambled siRNA (negative control, light grey). KIF11 knockdown is included as a positive control (black). MED19 depletion is highlighted in bold. A) Knockdown of Mediator subunits in MED19 LNCaP cells. B) Knockdown of Mediator subunits in LNCaP-abl cells. *p < 0.05; **p < 0.01; and ***p < 0.001. ns = not significant. Statistics denote comparison to scrambled siRNA. There is no statistically significant difference in growth between MED19 depletion and MED1 depletion in MED19 LNCaP cells or in LNCaP-abl cells. There is no statistically significant difference in growth between MED19 depletion and MED26/MED4/MED18/CDK19/MED12/MED27 depletion in MED19 LNCaP cells. (PDF) [file pgen.1008540.s019.pdf]

S20 Fig

A

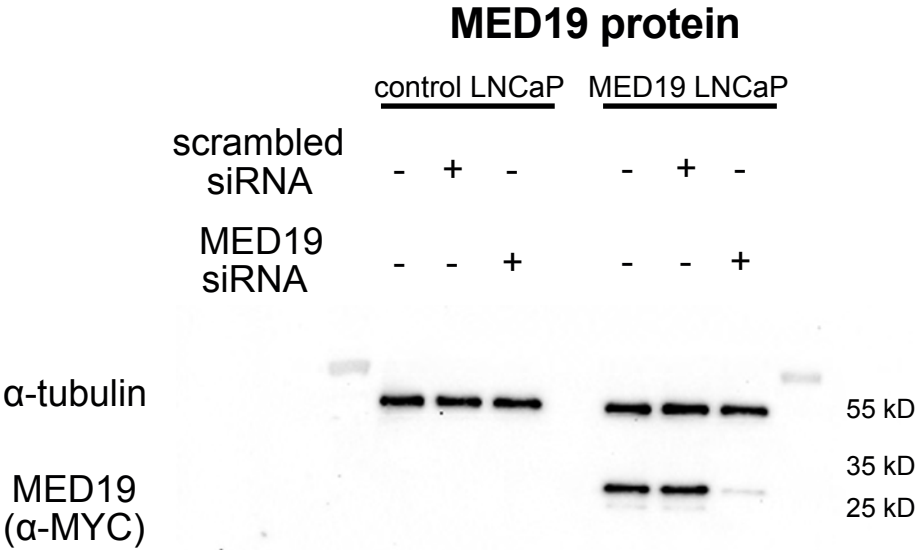

B

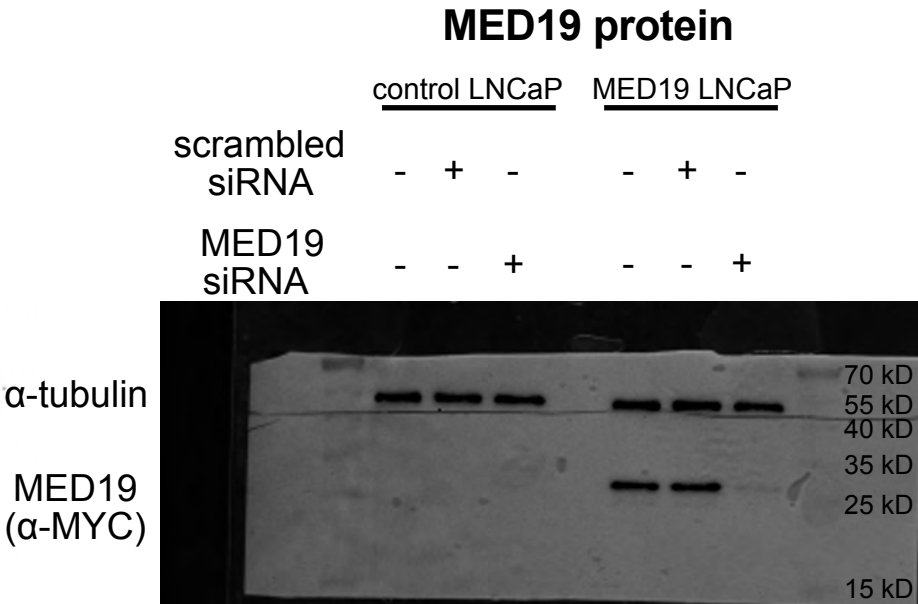

Supplement: S20 Fig — A) Full western blot from S1 Fig. B) Membrane overlay of full western blot from S1 Fig. (PDF) [file pgen.1008540.s020.pdf]
