# Supplementary material for: MED19 alters AR occupancy and gene expression in prostate cancer cells, driving MAOA expression and growth under low androgen
Source: PLoS Genet. 2021 Jan 29;17(1):e1008540. doi: 10.1371/journal.pgen.1008540 (PMC7875385; doi:10.1371/journal.pgen.1008540)
Supplement: S4 Fig — Immunohistochemistry of formalin-fixed, paraffin-embedded tissue sections from control MSC and MED19 MSC using antibodies against A) phospho-AKT1 Ser473 (pAKT) (Cell Signaling Cat. #4060, 1:100 dilution), B) phospho-p44/p42 ERK1/2 (pERK) (Cell Signaling Cat. #4376, 1:500 dilution), C) Ki-67 (BD Cat. #550609, 1:50 dilution), and D) AR (AR N-20, Santa Cruz Cat. #sc-816, 1:500 dilution). White arrow shows a cluster of cells with strong pERK staining in a tissue section from a MED19 MSC tumor. (PDF) [file pgen.1008540.s004.pdf]

S4 Fig

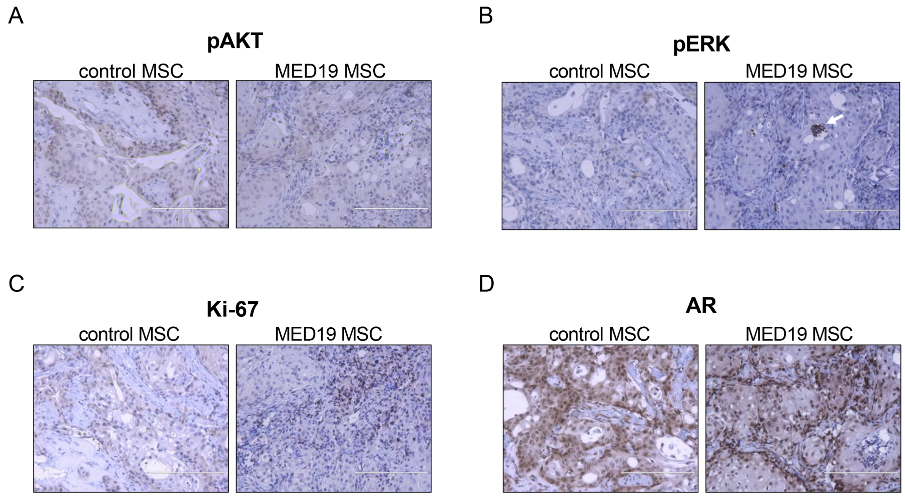
A
pAKT
control MSC
MED19 MSC
B
pERK
control MSC
MED19 MSC
C
Ki-67
control MSC
MED19 MSC
D
AR
control MSC
MED19 MSC
