# Supplementary material for: MED19 alters AR occupancy and gene expression in prostate cancer cells, driving MAOA expression and growth under low androgen
Source: PLoS Genet. 2021 Jan 29;17(1):e1008540. doi: 10.1371/journal.pgen.1008540 (PMC7875385; doi:10.1371/journal.pgen.1008540)
Supplement: S7 Fig — MED19 LNCaP cells and control LNCaP cells were cultured under androgen deprivation for 3 days and treated for 16 h with ethanol vehicle or 10 nM R1881. RNA was extracted and mRNA measured by qPCR for the AR target genes indicated (fold change expression normalized to RPL19 with target gene mRNA expression in vehicle-treated control LNCaP cells set as “1”). Experiment was performed in biological triplicate, with representative results shown. *p < 0.05; **p < 0.01; and ***p < 0.001. ns = not significant. (PDF) [file pgen.1008540.s007.pdf]

S7 Fig

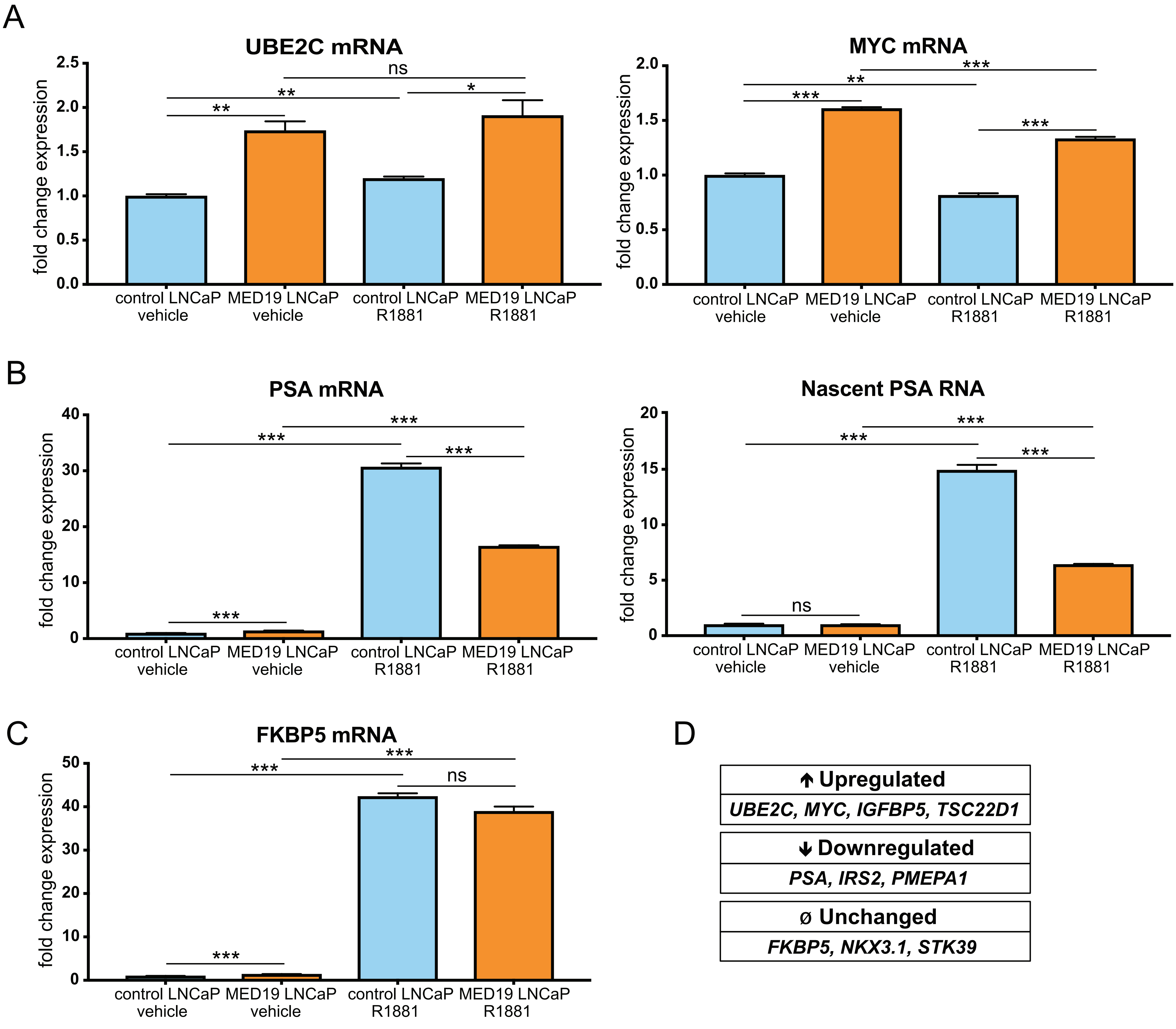
A
UBE2C mRNA
fold change expression
0.0
0.5
1.0
1.5
2.0
2.5
ns
**
*
**
control LNCaP vehicle
MED19 LNCaP vehicle
control LNCaP R1881
MED19 LNCaP R1881
MYC mRNA
fold change expression
0.0
0.5
1.0
1.5
2.0
***
**
***
***
control LNCaP vehicle
MED19 LNCaP vehicle
control LNCaP R1881
MED19 LNCaP R1881
B
PSA mRNA
fold change expression
0
10
20
30
40
***
***
***
***
control LNCaP vehicle
MED19 LNCaP vehicle
control LNCaP R1881
MED19 LNCaP R1881
Nascent PSA RNA
fold change expression
0
5
10
15
20
***
***
***
ns
control LNCaP vehicle
MED19 LNCaP vehicle
control LNCaP R1881
MED19 LNCaP R1881
C
FKBP5 mRNA
fold change expression
0
10
20
30
40
50
***
***
ns
***
control LNCaP vehicle
MED19 LNCaP vehicle
control LNCaP R1881
MED19 LNCaP R1881
D
Upregulated
UBE2C, MYC, IGFBP5, TSC22D1
Downregulated
PSA, IRS2, PMEPA1
ø Unchanged
FKBP5, NKX3.1, STK39
