# Supplementary material for: MED19 alters AR occupancy and gene expression in prostate cancer cells, driving MAOA expression and growth under low androgen
Source: PLoS Genet. 2021 Jan 29;17(1):e1008540. doi: 10.1371/journal.pgen.1008540 (PMC7875385; doi:10.1371/journal.pgen.1008540)

S11 Fig

A

LRRTM3 mRNA expression

RNA-seq

LRRTM3	Vehicle	R1881
Fold Change with MED19 overexpression	4.46	1.60
	Control LNCaP	MED19 LNCaP
Fold Change with R1881 treatment	-4.48	-15.43

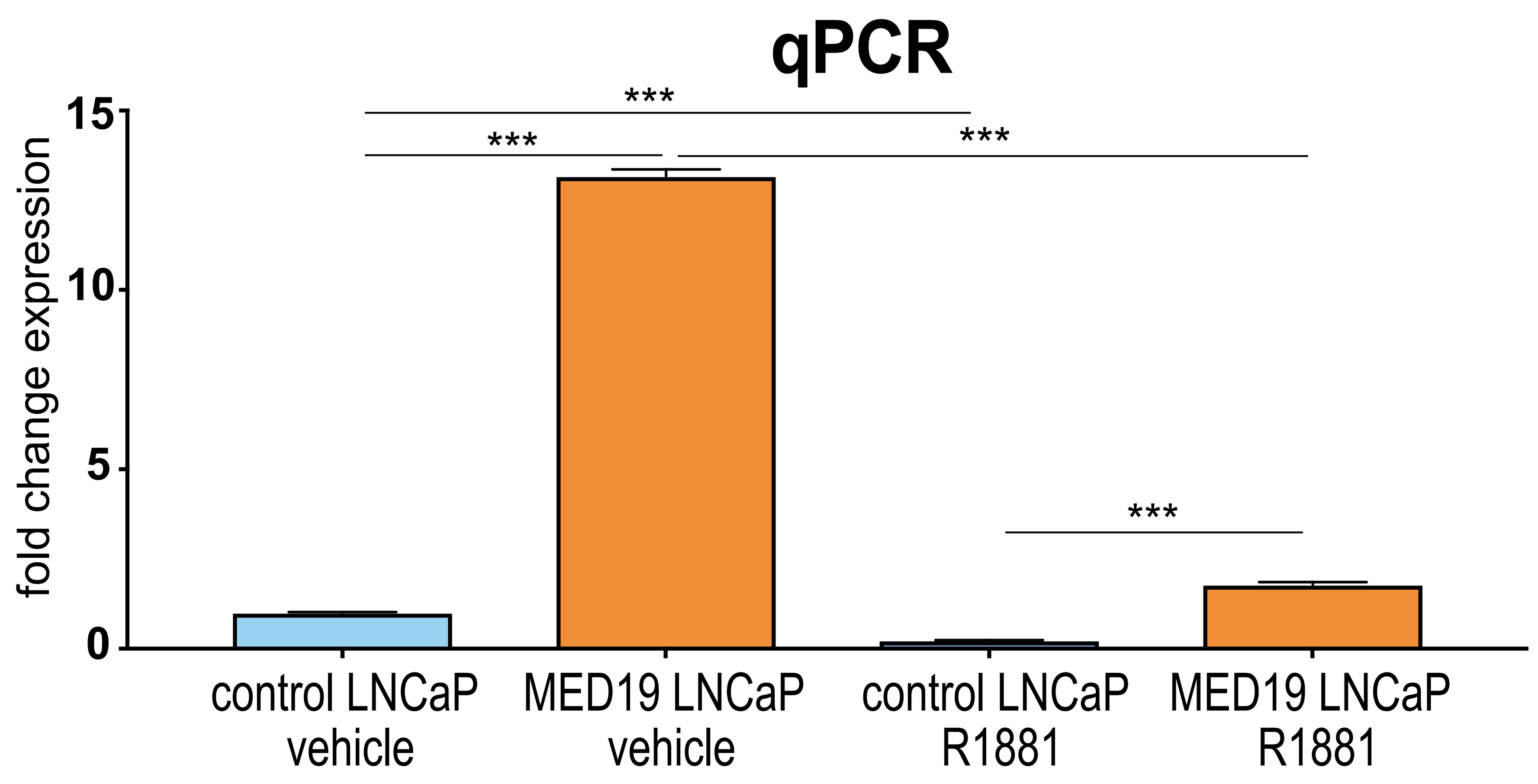

B

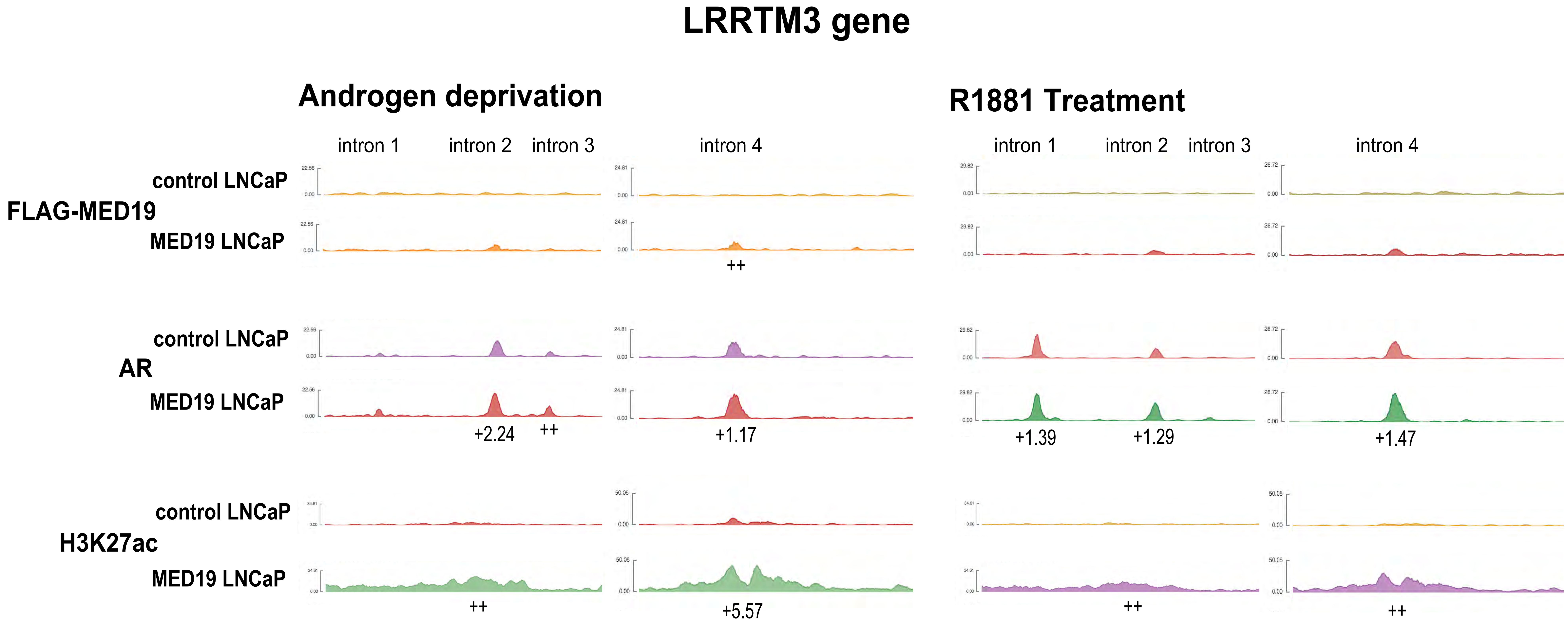

C

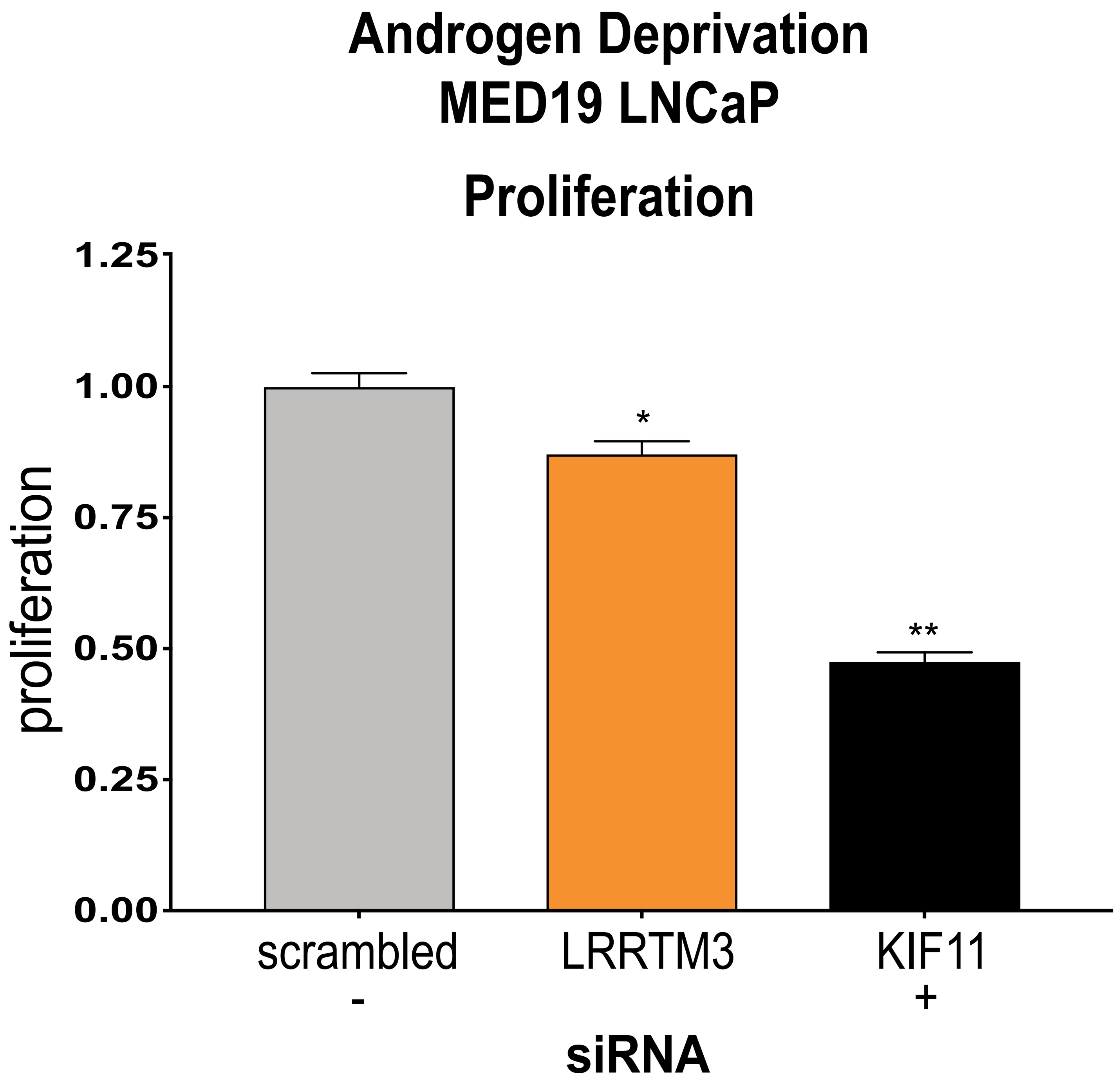

Supplement: S11 Fig — MED19 LNCaP cells and control LNCaP cells were cultured under androgen deprivation for 3 days and treated with ethanol vehicle or R1881 (10 nM 16 hours for RNA-seq; 100 nM 4 hours for ChIP-seq). RNA-seq and ChIP-seq for FLAG-MED19, AR, and H3K27ac were performed in biological triplicate, with the exception of ChIP-seq for AR in control LNCaP cells + R1881, where one sample was excluded from the analyses because of low signal. A) Fold change mRNA expression from RNA-seq and qPCR validation of changes in LRRTM3 mRNA expression (performed in biological triplicate, representative results shown; fold change expression normalized to RPL19 with LRRTM3 mRNA expression in vehicle-treated control LNCaP cells set as “1”). Greater fold changes by qPCR likely due to low abundance (raw counts in RNA-seq) of LRRTM3 in control LNCaP cells. B) ChIP-seq tracks (representative results) for FLAG-MED19, AR, and H3K27ac for androgen deprivation or R1881 treatment are shown for intronic regions of LRRTM3. Fold change (up (+) or down (-)) in occupancy scores for MED19 LNCaP cells compared to control LNCaP cells shown for each peak (see S6 Table for all occupancy scores). ++ indicates positive occupancy score in MED19 LNCaP cells and a score of zero in control LNCaP cells;—indicates an occupancy score of zero in MED19 LNCaP cells and a positive score in control LNCaP cells. C) LRRTM3 was depleted by siRNA and proliferation of MED19 LNCaP cells in androgen-depleted media was evaluated after 7 days, normalized to proliferation with scrambled siRNA (negative control, light grey). KIF11 knockdown is included as a positive control (black). Experiment was performed in biological duplicate, with representative results shown. *p < 0.05; **p < 0.01; and ***p < 0.001. (PDF) [file pgen.1008540.s011.pdf]
