# Supplementary material for: MED19 alters AR occupancy and gene expression in prostate cancer cells, driving MAOA expression and growth under low androgen
Source: PLoS Genet. 2021 Jan 29;17(1):e1008540. doi: 10.1371/journal.pgen.1008540 (PMC7875385; doi:10.1371/journal.pgen.1008540)

# S12 Fig

A

## MAST4 mRNA expression

### RNA-seq

| MAST4 | Vehicle | R1881 |
|---|---|---|
| Fold Change with MED19 overexpression | -2.37 | -2.29 |
| | Control LNCaP | MED19 LNCaP |
| Fold Change with R1881 treatment | -1.65 | -1.41 |

### qPCR

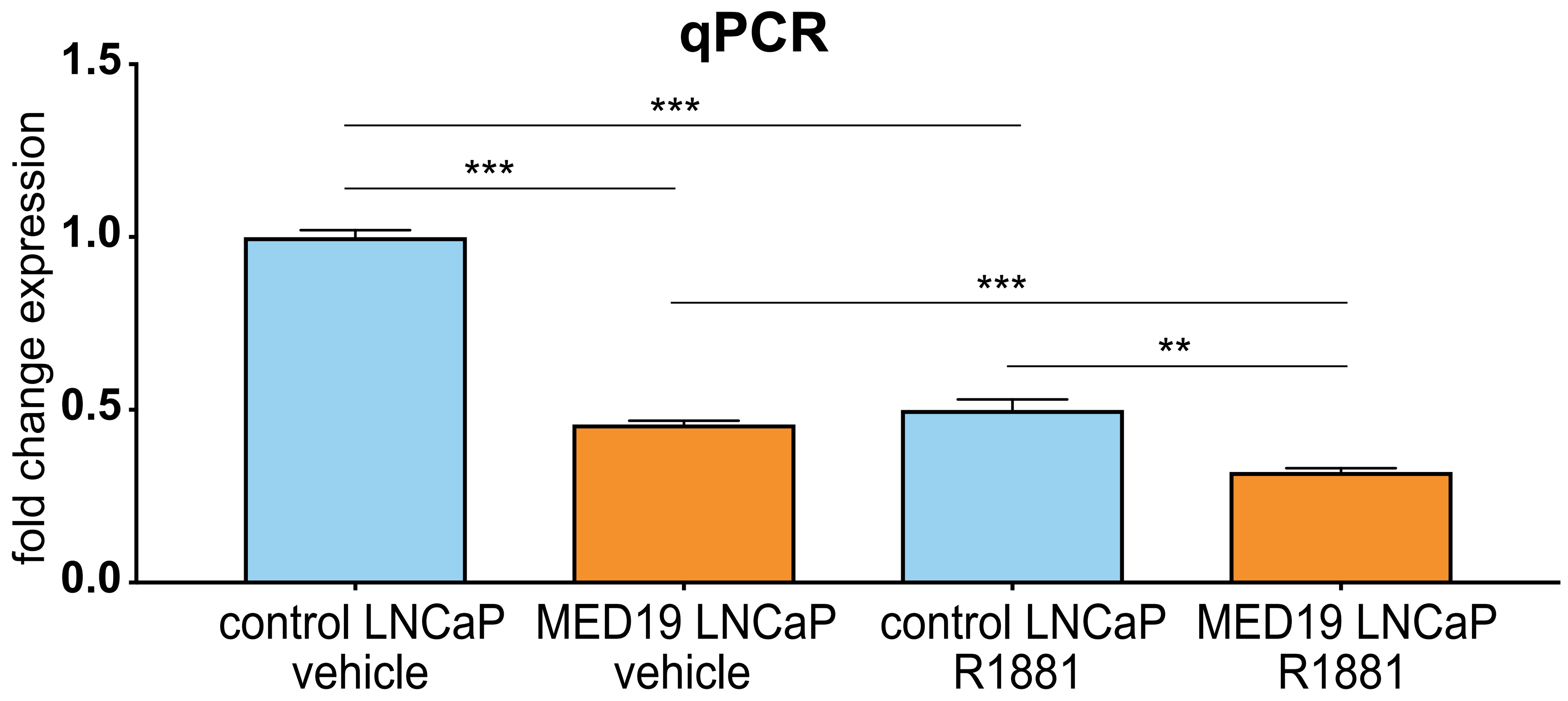


B

## MAST4 gene

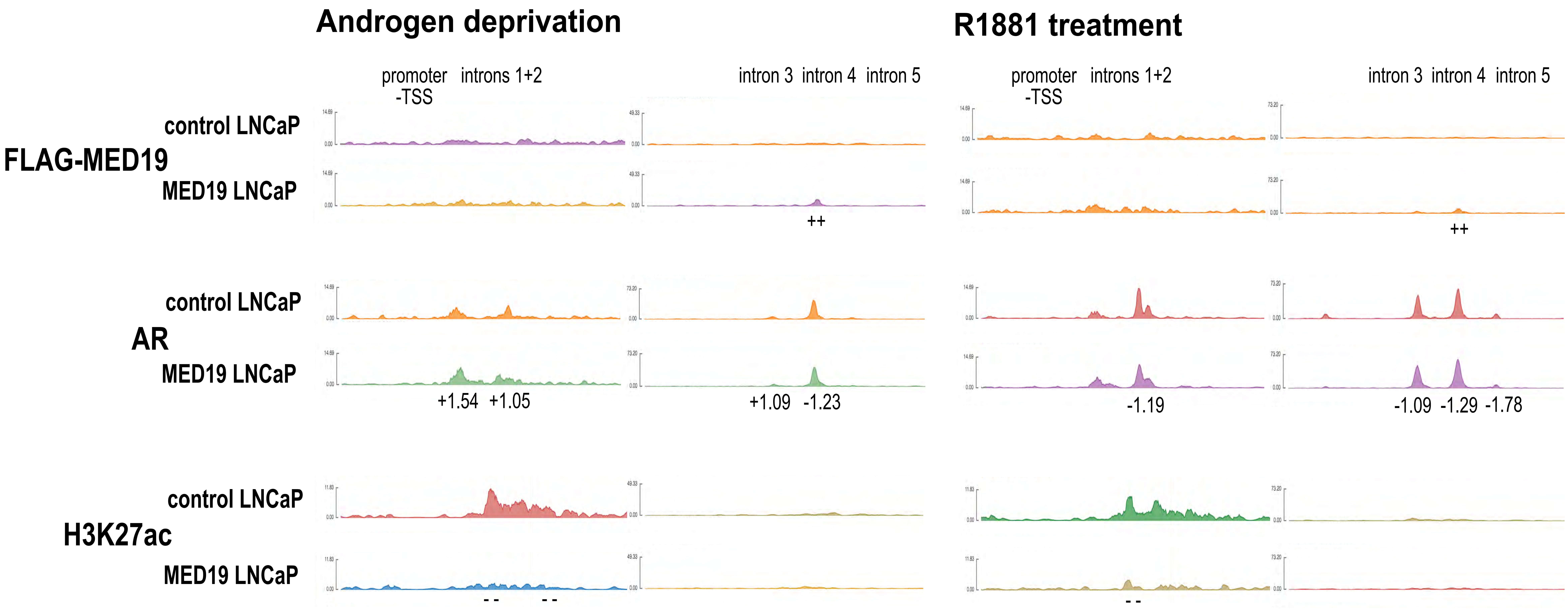

Supplement: S12 Fig — MED19 LNCaP cells and control LNCaP cells were cultured under androgen deprivation for 3 days and treated with ethanol vehicle or R1881 (10 nM 16 hours for RNA-seq; 100 nM 4 hours for ChIP-seq). RNA-seq and ChIP-seq for FLAG-MED19, AR, and H3K27ac were performed and in biological triplicate, with the exception of ChIP-seq for AR in control LNCaP cells + R1881, where one sample was excluded from the analyses because of low signal. A) Fold change mRNA expression from RNA-seq and qPCR validation of changes in MAST4 mRNA expression (performed in biological triplicate, representative results shown; fold change expression normalized to RPL19 with MAST4 mRNA expression in vehicle-treated control LNCaP cells set as “1”). *p < 0.05; **p < 0.01; and ***p < 0.001. B) ChIP-seq tracks (representative results) for FLAG-MED19, AR, and H3K27ac for androgen deprivation or R1881 treatment are shown for promoter and intronic regions of MAST4. Fold change (up (+) or down (-)) in occupancy scores for MED19 LNCaP cells compared to control LNCaP cells shown for each peak (see S6 Table for all occupancy scores). ++ indicates positive occupancy score in MED19 LNCaP cells and a score of zero in control LNCaP cells;—indicates an occupancy score of zero in MED19 LNCaP cells and a positive score in control LNCaP cells. (PDF) [file pgen.1008540.s012.pdf]
