# Supplementary material for: MED19 alters AR occupancy and gene expression in prostate cancer cells, driving MAOA expression and growth under low androgen
Source: PLoS Genet. 2021 Jan 29;17(1):e1008540. doi: 10.1371/journal.pgen.1008540 (PMC7875385; doi:10.1371/journal.pgen.1008540)
Supplement: S14 Fig — MED19 LNCaP cells and control LNCaP cells were cultured under androgen deprivation for 3 days and treated with ethanol vehicle or 100 nM R1881 for 4 hours. ChIP-seq for FLAG-MED19, AR, and H3K27ac was performed in biological triplicate, with the exception of ChIP-seq for AR in control LNCaP cells + R1881, where one sample was excluded from the analyses because of low signal. A) Top 10 enriched transcription factor motifs under androgen deprivation, associated with MED19 sites in MED19 LNCaP cells (top), MED19 and AR occupied sites in MED19 LNCaP cells (middle top), AR sites in MED19 LNCaP cells (middle bottom), and AR sites in control LNCaP cells (bottom). B) Top 10 enriched transcription factor motifs with R1881 treatment, associated with MED19 sites in MED19 LNCaP cells (top), MED19 and AR occupied sites in MED19 LNCaP cells (middle top), AR sites in MED19 LNCaP cells (middle bottom), and AR sites in control LNCaP cells (bottom). (PDF) [file pgen.1008540.s014.pdf]

S14 Fig

A

Androgen Deprivation

Top 10 motifs associated with MED19 in MED19 LNCaP cells

Motif	Match	% peaks with motif (% background)	p-value
	**FOXM1**	43.29% (13.49%)	1E-1241
	**FOXA1**	43.54% (14.01%)	1E-1199
	FOX:Ebox	35.08% (9.44%)	1E-1144
	FOXA2	33.99% (9.15%)	1E-1100
	FOXA3	21.35% (3.88%)	1E-966
	FOXK1	32.13% (12.63%)	1E-603
	FOXL2	29.98% (11.72%)	1E-559
	FOXF1	31.62% (13.02%)	1E-544
	FOXO3	25.90% (9.31%)	1E-540
	HOXB13	30.52% (12.80%)	1E-503

Top 10 motifs associated with AR+MED19 in MED19 LNCaP cells

Motif	Match	% peaks with motif (% background)	p-value
	**FOXM1**	40.33% (13.35%)	1E-1010
	**FOXA1**	40.76% (13.78%)	1E-993
	FOX:Ebox	32.26% (9.05%)	1E-951
	FOXA2	31.34% (8.75%)	1E-924
	FOXA3	19.06% (3.69%)	1E-778
	FOXK1	30.29% (12.54%)	1E-493
	FOXO3	24.30% (8.98%)	1E-464
	FOXF1	30.09% (12.99%)	1E-452
	FOXL2	28.00% (11.56%)	1E-451
	HOXB13	29.04% (12.61%)	1E-427

Top 10 motifs associated with AR in MED19 LNCaP cells

Motif	Match	% peaks with motif (% background)	p-value
	**FOXM1**	48.17% (16.53%)	1E-11572
	**FOXA1**	48.74% (17.25%)	1E-11246
	FOXA2	36.63% (11.04%)	1E-9671
	FOX:Ebox	36.01% (11.06%)	1E-9245
	FOXA3	22.14% (4.82%)	1E-7817
	FOXK1	36.20% (15.49%)	1E-5519
	FOXF1	36.89% (16.43%)	1E-5231
	FOXL2	34.24% (14.63%)	1E-5155
	FOXO3	29.29% (11.46%)	1E-5002
	FOXP1	19.70% (6.37%)	1E-4272

Top 10 motifs associated with AR in control LNCaP cells

Motif	Match	% peaks with motif (% background)	p-value
	**FOXM1**	51.95% (17.80%)	1E-13806
	**FOXA1**	52.59% (18.46%)	1E-13582
	FOXA2	38.97% (11.88%)	1E-11052
	FOX:Ebox	38.13% (11.66%)	1E-10708
	FOXA3	23.52% (5.01%)	1E-9172
	FOXK1	38.55% (16.81%)	1E-6191
	FOXF1	39.71% (17.86%)	1E-6058
	FOXL2	36.74% (15.91%)	1E-5898
	FOXO3	31.17% (12.41%)	1E-5611
	FOXP1	20.79% (6.89%)	1E-4704

B

R1881 Treatment

Top 10 motifs associated with MED19 in MED19 LNCaP cells

Motif	Match	% peaks with motif (% background)	p-value
	**FOXM1**	33.39% (9.94%)	1E-1241
	**FOXA1**	33.58% (10.08%)	1E-1199
	FOXA2	26.96% (6.94%)	1E-1144
	FOX:Ebox	28.15% (8.36%)	1E-1100
	FOXA3	16.16% (2.83%)	1E-966
	FOXK1	24.82% (9.61%)	1E-603
	FOXL2	22.45% (8.28%)	1E-559
	FOXO3	20.23% (6.97%)	1E-544
	FOXF1	23.43% (8.98%)	1E-540
	FOXP1	14.15% (3.98%)	1E-503

Top 10 motifs associated with AR+MED19 in MED19 LNCaP cells

Motif	Match	% peaks with motif (% background)	p-value
	**FOXM1**	40.21% (12.65%)	1E-1256
	**FOXA1**	39.90% (12.54%)	1E-1245
	FOXA2	31.56% (8.66%)	1E-1111
	FOX:Ebox	32.77% (9.72%)	1E-1054
	FOXA3	18.40% (3.57%)	1E-871
	FOXK1	29.61% (12.11%)	1E-572
	FOXL2	27.44% (10.73%)	1E-566
	FOXO3	24.14% (8.91%)	1E-537
	FOXF1	28.58% (11.83%)	1E-537
	FOXP1	16.54% (4.94%)	1E-482

Top 10 motifs associated with AR in MED19 LNCaP cells

Motif	Match	% peaks with motif (% background)	p-value
	**FOXM1**	49.90% (17.77%)	1E-17196
	**FOXA1**	54.78% (21.74%)	1E-16623
	FOXA2	37.10% (12.13%)	1E-13161
	FOX:Ebox	36.65% (12.13%)	1E-12620
	FOXA3	21.61% (5.14%)	1E-10356
	**ARE**	11.30% (1.64%)	1E-8151
	GRE	12.06% (1.96%)	1E-7944
	FOXK1	37.27% (16.98%)	1E-7551
	FOXL2	34.99% (15.61%)	1E-7265
	FOXF1	37.66% (17.62%)	1E-7238

Top 10 motifs associated with AR in control LNCaP cells

Motif	Match	% peaks with motif (% background)	p-value
	**FOXM1**	51.99% (18.22%)	1E-18076
	**FOXA1**	56.90% (22.30%)	1E-17426
	FOXA2	38.34% (12.41%)	1E-17287
	FOX:Ebox	37.92% (12.55%)	1E-13500
	FOXA3	22.39% (5.24%)	1E-12900
	FOXK1	38.41% (17.26%)	1E-10640
	FOXF1	38.98% (17.90%)	1E-7835
	FOXL2	36.13% (16.02%)	1E-7643
	**ARE**	10.70% (1.58%)	1E-7435
	GRE	11.53% (1.94%)	1E-7354
