# Supplementary material for: MED19 alters AR occupancy and gene expression in prostate cancer cells, driving MAOA expression and growth under low androgen
Source: PLoS Genet. 2021 Jan 29;17(1):e1008540. doi: 10.1371/journal.pgen.1008540 (PMC7875385; doi:10.1371/journal.pgen.1008540)
Supplement: S15 Fig — A) Top 10 enriched transcription factor motifs with R1881 treatment associated with sites of AR and MED19 occupancy in MED19 LNCaP cells where AR is present only in MED19 LNCaP cells, with SP1 as the top associated transcription factor. B) Gene changes associated with ELK1 knockdown and AR knockdown from Enrichr Transcription Factor Perturbation, compared to MED19 overexpression under androgen deprivation from the RNA-seq study. C) SRF knockdown is the top hit from Enrichr Transcription Factor Perturbation, associated with genes upregulated by MED19 overexpression with R1881 treatment from the RNA-seq study (top); corresponding genes changes associated with SRF knockdown compared to MED19 overexpression (bottom). (PDF) [file pgen.1008540.s015.pdf]

S15 Fig

A

R1881 Treatment

Top 10 motifs associated with MED19+AR only in MED19 LNCaP cells

Motif	Match	% peaks with motif (% background)	p-value
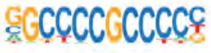	**SP1**	33.59% (16.49%)	1E-14
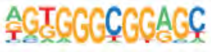	SP5	51.90% (32.58%)	1E-14
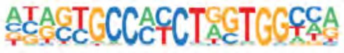	CTCF	8.16% (1.62%)	1E-12
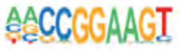	ETS	15.84% (6.34%)	1E-11
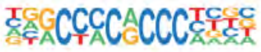	KLF3	29.22% (15.98%)	1E-11
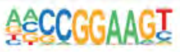	ELF1	22.11% (10.91%)	1E-9
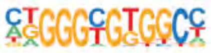	KLF6	44.31% (29.37%)	1E-9
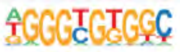	KLF5	50.76% (35.50%)	1E-9
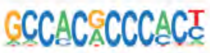	KLF9	22.96% (11.89%)	1E-8
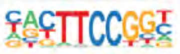	ELK1	23.34% (12.21%)	1E-8

B

Androgen Deprivation

RNA-seq
Genes Upregulated with MED19 OE

Enrichr TF Perturbation Analysis

Gene	ELK1 KD	AR KD	MED19 OE
SYT4	down	down	up
PLA2G2A	down	down	up
ATP1B1	down	down	up
NIPSNAP3A	down	down	up
**MAOA**	**down**	**down**	**up**
SLC44A1	down	down	up
BHCE	down	down	up
ADAM2	down	down	up
SQOR	down		up
TMSB4X	down		up
CAMK2N1	down		up
GALNT3	down		up
PKIB		down	up
MANEA		down	up
KCNN2		down	up

C

R1881 Treatment

RNA-seq
Genes Upregulated with MED19 OE

Enrichr TF Perturbation Analysis

Perturbation	p-value
SRF knockdown	6.15E-10

Gene	SRF KD	MED19 OE
ABCA12	down	up
RAB27A	down	up
GLYATL2	down	up
RBM24	down	up
SYT4	down	up
TMEM140	down	up
GNAI1	down	up
ZNF385B	down	up
GPR158	down	up
SEMA3C	down	up
SSFA2	down	up
DDIT4L	down	up
DPP4	down	up
