# Supplementary material for: MED19 alters AR occupancy and gene expression in prostate cancer cells, driving MAOA expression and growth under low androgen
Source: PLoS Genet. 2021 Jan 29;17(1):e1008540. doi: 10.1371/journal.pgen.1008540 (PMC7875385; doi:10.1371/journal.pgen.1008540)
Supplement: S16 Fig — MED19 LNCaP cells and control LNCaP cells were cultured under androgen deprivation for 3 days and treated with ethanol vehicle or 100 nM R1881 for 4 hours. ChIP-seq for FLAG-MED19, AR, and H3K27ac was performed in biological triplicate, with the exception of ChIP-seq for AR in control LNCaP cells + R1881, where one sample was excluded from the analyses because of low signal. A) Top 10 enriched transcription factor motifs associated with AR sites in control LNCaP cells for R1881 vs. vehicle treatment, with enrichment of AR-related motifs in response to R1881 treatment. B) Top 10 enriched transcription factor motifs associated with AR sites in MED19 LNCaP cells for R1881 vs. vehicle treatment, with enrichment of AR-related motifs in response to R1881 treatment. C) Top 10 enriched transcription factor motifs associated with MED19 sites in MED19 LNCaP cells for R1881 vs. vehicle treatment, with enrichment of AR-related motifs in response to R1881 treatment. (PDF) [file pgen.1008540.s016.pdf]

S16 Fig

A

control LNCaP
Vehicle vs R1881 Treatment

Top 10 motifs associated with AR with vehicle and R1881 treatment

Motif	Match	% peaks with motif (% background)	p-value
	FOXM1	55.03% (18.33%)	1E-11895
	FOXA1	60.08% (22.52%)	1E-11481
	FOXA2	41.36% (12.22%)	1E-9459
	FOX:Ebox	40.67% (12.17%)	1E-9118
	FOXA3	24.96% (5.23%)	1E-7601
	FOXK1	40.52% (16.96%)	1E-5459
	FOXF1	41.39% (18.05%)	1E-5200
	FOXL2	38.49% (16.27%)	1E-5010
	FOXO3	32.87% (12.50%)	1E-4941
	FOXP1	22.10% (6.96%)	1E-4141

Additional AR-related motifs

Motif	Match	% peaks with motif (% background)	p-value
	FOXA1:AR	7.19% (1.55%)	1E-1966
	AR-halfsite	44.02% (32.67%)	1E-988
	ARE	5.14% (1.57%)	1E-917

Top 10 motifs associated with AR with R1881 treatment only

Motif	Match	% peaks with motif (% background)	p-value
	GRE	19.25% (2.08%)	1E-7453
	ARE	18.01% (1.74%)	1E-7445
	FOXM1	48.28% (18.18%)	1E-6399
	PR	54.09% (22.52%)	1E-6381
	PGR	20.00% (3.01%)	1E-6144
	FOXA1	53.07% (22.23%)	1E-6137
	FOXA1:AR	13.52% (1.54%)	1E-4958
	FOXA2	34.66% (12.85%)	1E-4255
	FOX:Ebox	34.48% (12.99%)	1E-4114
	FOXA3	19.27% (5.46%)	1E-3155

Additional AR-related motifs

Motif	Match	% peaks with motif (% background)	p-value
	AR-halfsite	54.60% (34.74%)	1E-2244

Top 10 motifs associated with AR with vehicle treatment only

Motif	Match	% peaks with motif (% background)	p-value
	FOXM1	37.62% (14.93%)	1E-1686
	FOXA1	38.60% (15.79%)	1E-1652
	FOXA2	28.03% (9.72%)	1E-1463
	FOX:Ebox	26.53% (9.62%)	1E-1279
	FOXA3	16.38% (4.18%)	1E-1201
	HOXB13	33.53% (15.25%)	1E-1124
	FOXK1	29.44% (14.19%)	1E-841
	FOXF1	31.25% (15.68%)	1E-823
	FOXL2	28.51% (13.71%)	1E-813
	FOXO3	23.59% (10.32%)	1E-795

Additional AR-related motifs

Motif	Match	% peaks with motif (% background)	p-value
	AR-halfsite	31.67% (29.45%)	1E-14

B

MED19 LNCaP
Vehicle vs R1881 Treatment

Top 10 motifs associated with AR with vehicle and R1881 treatment

Motif	Match	% peaks with motif (% background)	p-value
	FOXM1	51.88% (17.76%)	1E-10127
	FOXA1	52.47% (18.39%)	1E-9955
	FOXA2	39.38% (11.92%)	1E-8287
	FOX:Ebox	38.72% (11.95%)	1E-7917
	FOXA3	23.80% (5.04%)	1E-6857
	FOXK1	38.74% (16.61%)	1E-4730
	FOXF1	39.36% (17.23%)	1E-4635
	FOXL2	36.56% (15.55%)	1E-4452
	FOXO3	31.46% (12.07%)	1E-4438
	FOXP1	21.19% (6.59%)	1E-3853

Additional AR-related motifs

Motif	Match	% peaks with motif (% background)	p-value
	FOXA1:AR	6.36% (1.49%)	1E-1531
	AR-halfsite	44.58% (32.51%)	1E-1073
	ARE	5.10% (1.54%)	1E-889

Top 10 motifs associated with AR with R1881 treatment only

Motif	Match	% peaks with motif (% background)	p-value
	ARE	18.16% (1.62%)	1E-8795
	GRE	19.31% (2.00%)	1E-8605
	PGR	20.07% (2.94%)	1E-7042
	FOXM1	47.49% (17.82%)	1E-7039
	PR	54.43% (23.20%)	1E-6914
	FOXA1	52.30% (22.00%)	1E-6674
	FOXA1:AR	13.07% (1.64%)	1E-5011
	FOXA2	34.35% (12.73%)	1E-4712
	FOX:Ebox	34.06% (13.10%)	1E-4385
	FOXA3	19.11% (5.32%)	1E-3577

Additional AR-related motifs

Motif	Match	% peaks with motif (% background)	p-value
	AR-halfsite	54.85% (32.51%)	1E-2517

Top 10 motifs associated with AR with vehicle treatment only

Motif	Match	% peaks with motif (% background)	p-value
	FOXM1	29.30% (11.86%)	1E-992
	FOXA1	29.83% (12.31%)	1E-979
	FOXA2	22.25% (8.06%)	1E-870
	FOX:Ebox	21.49% (8.16%)	1E-774
	FOXA3	12.81% (3.38%)	1E-739
	HOXB13	27.10% (12.78%)	1E-664
	FOXK1	23.54% (11.50%)	1E-517
	FOXO3	18.85% (8.37%)	1E-497
	NF1	10.32% (3.14%)	1E-489
	FOXF1	24.10% (12.24%)	1E-484

Additional AR-related motifs

Motif	Match	% peaks with motif (% background)	p-value
	AR-halfsite	31.58% (30.58%)	1E-3

C

MED19 LNCaP
Vehicle vs R1881 Treatment

Top 10 motifs associated with MED19 with vehicle and R1881 treatment

Motif	Match	% peaks with motif (% background)	p-value
	FOXA1	37.26% (10.72%)	1E-680
	FOXM1	36.74% (10.47%)	1E-676
	FOX:Ebox	31.78% (8.25%)	1E-634
	FOXA2	29.96% (7.31%)	1E-634
	FOXA3	18.61% (3.07%)	1E-542
	FOXK1	28.10% (10.06%)	1E-358
	FOXO3	22.59% (7.26%)	1E-327
	FOXL2	25.27% (9.03%)	1E-317
	FOXF1	26.05% (9.88%)	1E-298
	FOXK2	18.05% (5.49%)	1E-275

Additional AR-related motifs

Motif	Match	% peaks with motif (% background)	p-value
	FOXA1:AR	2.99% (0.90%)	1E-44
	AR-halfsite	39.84% (39.84%)	1E-24
	ARE	3.16% (1.53%)	1E-19

Top 10 motifs associated with MED19 with R1881 treatment only

Motif	Match	% peaks with motif (% background)	p-value
	FOXM1	31.58% (9.31%)	1E-850
	FOXA1	31.03% (9.14%)	1E-834
	FOXA2	25.22% (6.80%)	1E-732
	FOX:Ebox	25.93% (7.92%)	1E-644
	FOXA3	14.71% (2.81%)	1E-582
	GRE	11.00% (1.63%)	1E-532
	PGR	10.65% (1.59%)	1E-512
	ARE	10.44% (1.59%)	1E-494
	FOXA1:AR	7.43% (0.77%)	1E-461
	PR	34.49% (17.27%)	1E-378

Additional AR-related motifs

Motif	Match	% peaks with motif (% background)	p-value
	AR-halfsite	44.94% (33.78%)	1E-118

Top 10 motifs associated with MED19 with vehicle treatment only

Motif	Match	% peaks with motif (% background)	p-value
	FOXM1	53.18% (17.31%)	1E-609
	FOXA1	53.20% (18.35%)	1E-564
	FOX:Ebox	40.16% (10.76%)	1E-533
	FOXA2	39.90% (11.47%)	1E-487
	FOXA3	25.34% (4.86%)	1E-438
	HOXB13	40.23% (16.87%)	1E-281
	FOXK1	38.38% (16.49%)	1E-253
	FOXF1	40.35% (17.97%)	1E-252
	FOXL2	36.87% (15.82%)	1E-241
	FOXO3	31.10% (12.10%)	1E-232

Additional AR-related motifs

Motif	Match	% peaks with motif (% background)	p-value
	FOXA1:AR	2.06% (1.38%)	1E-3
