# Supplementary material for: MED19 alters AR occupancy and gene expression in prostate cancer cells, driving MAOA expression and growth under low androgen
Source: PLoS Genet. 2021 Jan 29;17(1):e1008540. doi: 10.1371/journal.pgen.1008540 (PMC7875385; doi:10.1371/journal.pgen.1008540)

S13 Fig

A

MAOA mRNA expression

RNA-seq

MAOA	Vehicle	R1881
Fold Change with MED19 overexpression	1.56	ns
	Control LNCaP	MED19 LNCaP
Fold Change with R1881 treatment	1.73	1.26

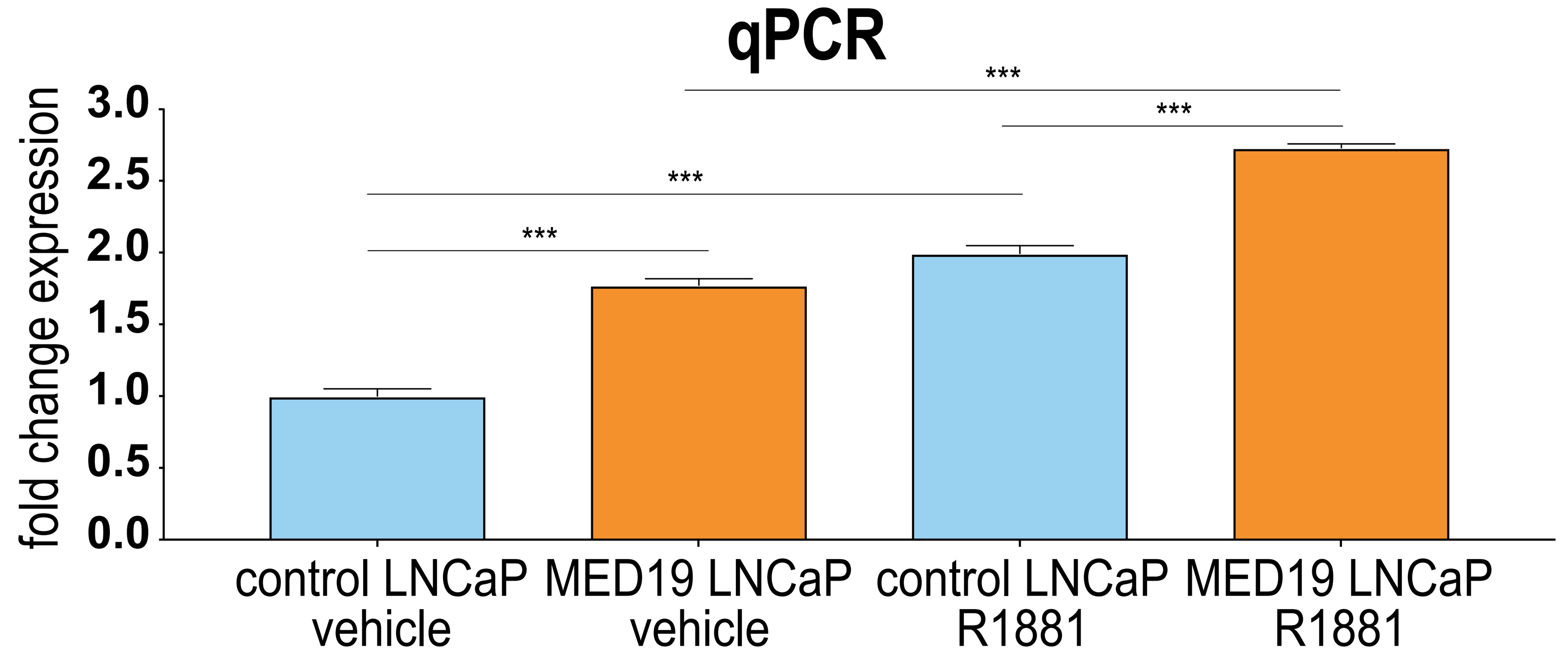

B

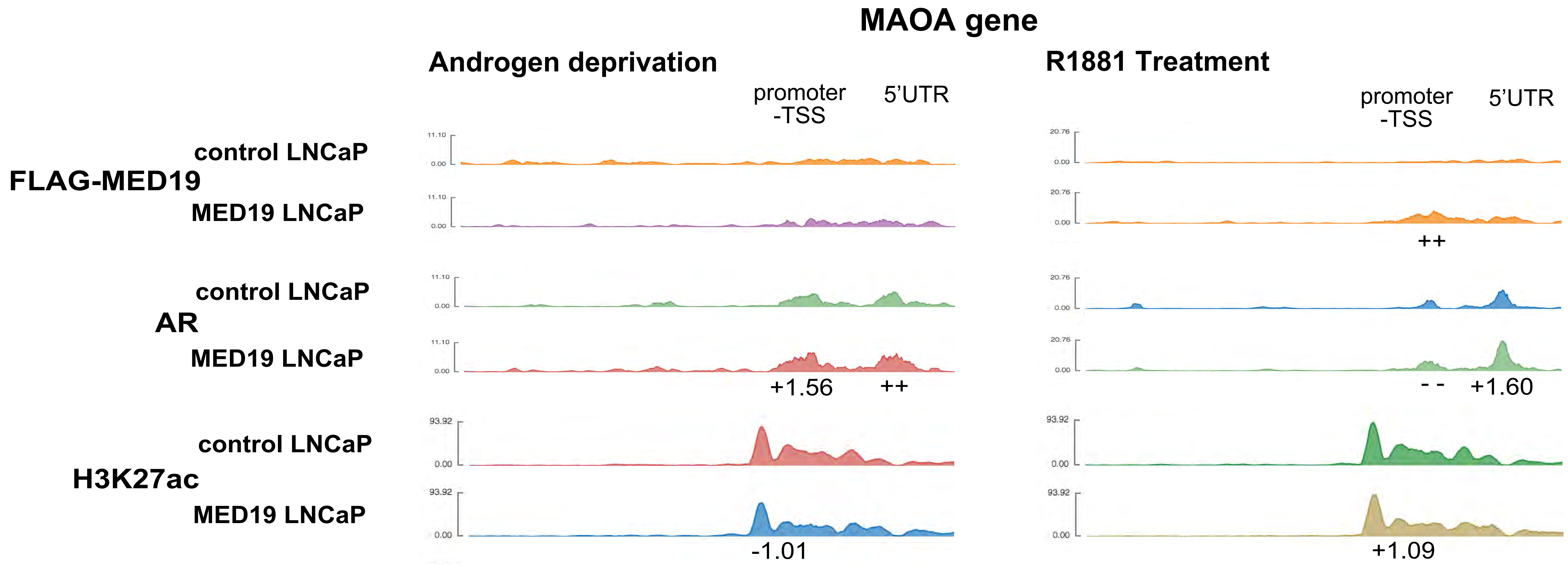

C

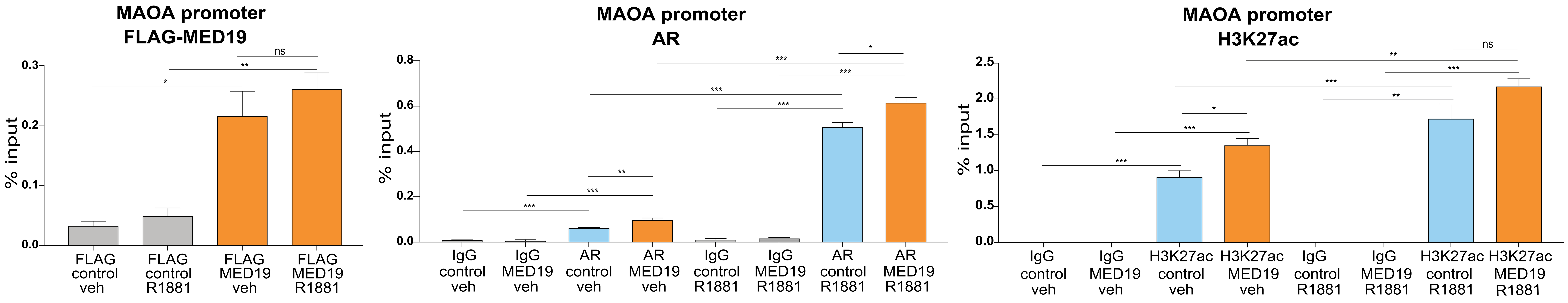

Supplement: S13 Fig — MED19 LNCaP cells and control LNCaP cells were cultured under androgen deprivation for 3 days and treated with ethanol vehicle or R1881 (10 nM 16 hours for RNA-seq; 100 nM 4 hours for ChIP-seq). RNA-seq and ChIP-seq for FLAG-MED19, AR, and H3K27ac were performed in biological triplicate, with the exception of ChIP-seq for AR in control LNCaP cells + R1881, where one sample was excluded from the analyses because of low signal. A) Fold change mRNA expression from RNA-seq and qPCR validation of changes in MAOA mRNA expression (performed in biological triplicate, representative results shown; fold change expression normalized to RPL19 with MAOA mRNA expression in vehicle-treated control LNCaP cells set as “1”). B) ChIP-seq tracks (representative results) for FLAG-MED19, AR, and H3K27ac for androgen deprivation or R1881 treatment are shown for the promoter region of MAOA. Fold change (up (+) or down (-)) in occupancy scores for MED19 LNCaP cells compared to control LNCaP cells shown for each peak (see S6 Table for all occupancy scores). ++ indicates positive occupancy score in MED19 LNCaP cells and a score of zero in control LNCaP cells;—indicates an occupancy score of zero in MED19 LNCaP cells and a positive score in control LNCaP cells. C) ChIP-qPCR for FLAG-MED19, AR, and H3K27ac at the MAOA promoter overlapping with published ARE. (Control–control LNCaP cells; MED19 –MED19 LNCaP cells; veh–vehicle treatment; R1881 –R1881 treatment). Experiment was performed in biological duplicate, with representative results shown. *p < 0.05; **p < 0.01; and ***p < 0.001. ns = not significant. (PDF) [file pgen.1008540.s013.pdf]
